# scLink: Inferring Sparse Gene Co-expression Networks from Single-cell Expression Data

**DOI:** 10.1016/j.gpb.2020.11.006

**Published:** 2021-07-10

**Authors:** Wei Vivian Li, Yanzeng Li

**Affiliations:** Department of Biostatistics and Epidemiology, Rutgers School of Public Health, Rutgers, The State University of New Jersey, Piscataway, NJ 08854, USA

**Keywords:** Gene co-expression network, Single-cell RNA sequencing, Network modeling, Robust correlation

## Abstract

A system-level understanding of the regulation and coordination mechanisms of gene expression is essential for studying the complexity of biological processes in health and disease. With the rapid development of **single-cell RNA sequencing** technologies, it is now possible to investigate gene interactions in a cell type-specific manner. Here we propose the scLink method, which uses statistical **network modeling** to understand the co-expression relationships among genes and construct sparse **gene co-expression networks** from single-cell gene expression data. We use both simulation and real data studies to demonstrate the advantages of scLink and its ability to improve single-cell gene network analysis. The scLink R package is available at https://github.com/Vivianstats/scLink.

## Introduction

Biological systems often involve tens of thousands of genes tightly regulated in complex and dynamic networks, which could change substantially among different tissue types, developmental stages, or cell states [1], [2]. Therefore, elucidating gene interactions in a network manner is crucial for understanding complex biological processes in human physiology and pathology. Identifying abnormal gene interactions in disease states makes it possible to reveal the biological and biochemical pathways relevant to disease mechanisms and therapeutic targets [3]. For instance, transcriptional dysregulation revealed by disease-associated gene interactions has been reported in various diseases including cancer [4], neurological disorders [5], and psychiatric disorders [6], leading to functional insights of transcriptome organization in disease progression.

In network analysis, genes are represented by nodes, and their relationships are depicted by directed or undirected edges between the nodes. The gene networks constructed from bulk tissue RNA sequencing (RNA-seq) data have played a key role in identifying genes responsible for similar biological functions, targets of transcriptional regulation, and regulators of disease-associated biological pathways [7], [8], [9]. However, the tissue-level networks can only describe the average gene-gene relationships across multiple biological samples, with the assumption that cells maintain the same regulatory mechanisms across diverse cell types [10]. Rapid advances of single-cell RNA sequencing (scRNA-seq) technologies have now made it possible to investigate gene networks across individual cells in a cell type-specific manner [11]. Functional networks constructed from scRNA-seq data have provided novel insights into the transcriptional regulation mechanisms underlying various biological processes, including cancer progression [12], immune system response [13], and embryonic development [14]. These studies have demonstrated the power of scRNA-seq analysis to reveal gene covariance structures in distinct cell types, identify genetic variants that can alter gene co-expression, and infer gene regulatory networks that can govern lineage decisions.

Even though exploratory analyses demonstrated the possibilities of constructing functional gene networks across single cells, both technical and biological complications present challenges to the genome-wide inference of gene dependencies from scRNA-seq data [15]. Due to technical molecular inefficiencies, a truly expressed gene may not be detected by scRNA-seq in some cells, and thus is represented by a false zero expression [16]. Meanwhile, stochastic gene expression process can also lead to zero expression representing biological variation. Therefore, scRNA-seq data are often much sparser than the bulk RNA-seq data, requiring new statistical and computational tools that could tackle the modeling challenges given the excessive zero counts. In bulk RNA-seq data analysis, gene network studies mostly rely on the Pearson or Spearman’s correlation coefficients to characterize the gene co-expression strength [17], [18]. However, these two measures cannot provide a robust estimation of gene co-expression given the sparse scRNA-seq data with substantial technical noises and biological heterogeneity [19], [20].

In light of the aforementioned problem, Iacono et al. [18] used the correlation between two genes’ differential expression patterns across cell types instead of gene expression levels to study gene regulatory network plasticity. However, most reconstruction methods of gene networks do not explicitly account for the sparsity issue. For example, PIDC uses partial information decomposition based on the multivariate information theory to quantify the statistical dependencies between genes and infer gene networks from scRNA-seq data [21]. GENIE3 decomposes the prediction of a gene network between p genes into p different regression problems, and uses tree-based ensemble methods to infer the edges between genes [22]. It has been shown to have a competitive performance on bulk data [23] and has also been applied to single-cell data for gene network inference [24]. In addition to methods that are purely based on gene expression data, there are also single-cell methods developed to infer direct gene regulatory relationships instead of statistical dependencies [25]. To infer the direct gene interactions, these methods typically require external information, such as time points or pseudo-time order of the cells [26], [27] and known transcription factors (TFs) [24].

Despite being an active research area, accurate inference of functional gene networks from single-cell gene expression data remains a challenge [15], [25]. In this study, we propose a new method named scLink to better characterize the statistical dependencies between genes in single cells and improve the construction of gene co-expression networks based on a new co-expression measure. In summary, scLink has the following key features and advantages. First, it proposes a robust estimator for measuring gene co-expression strength, built upon our previous work on improving the quality of single-cell gene expression data [28]. Instead of using all the observed read counts to measure the association between two genes, scLink relies on the cells in which both genes are accurately measured with high confidence. Second, scLink adapts the Gaussian graphical model [29] to distinguish direct associations between genes from indirect ones and leads to sparse networks. Under this framework, the absence of an edge between two genes indicates the independence of these two genes conditioned on all other genes. Gaussian graphical models have been widely used to infer biological networks from genomic data. They have revealed cancer type-specific gene interactions that potentially contribute to cancer development and progression [30], [31], [32]. Third, scLink uses a penalized likelihood approach to identify relatively sparse gene networks in a data-adaptive manner, adjusting the penalty strength on each edge based on the observed co-expression strength in single cells. Our approach is a modified version of the graphical lasso method [33] to improve the identification of edges using single-cell data. Fourth, scLink provides a unique method to construct sparse gene co-expression networks, increasing the interpretability of single-cell network analysis. We show that by combining the aforementioned features, scLink could enable more robust quantification of gene co-expression relationships, more accurate construction of gene co-expression networks, and better identification of functional gene modules which provide insights into cell type-specific transcriptional regulatory mechanisms and molecular pathways.

## Method

### Overview of the scLink method

**Figure 1:**
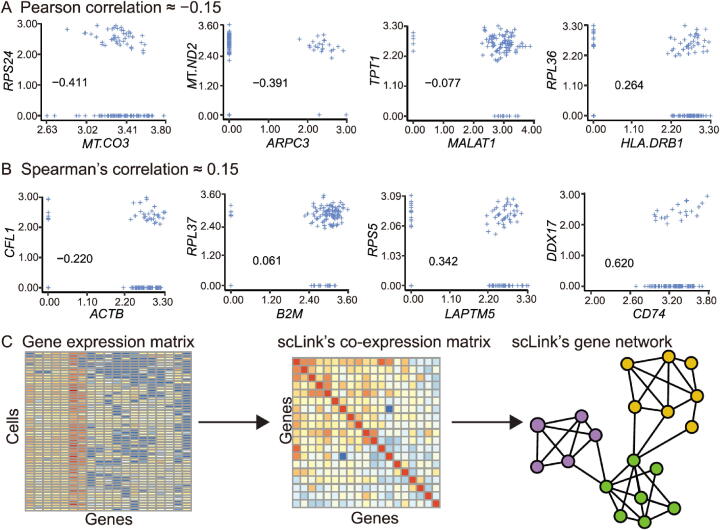
**The motivation and workflow of the scLink method. A.** Example gene pairs with similar Pearson correlation coefficients in B cells. **B.** Example gene pairs with similar Spearman’s correlation coefficients in B cells. The scatter plots show the log_10_-transformed gene expression levels. The Pearson or Spearman’s correlation coefficients calculated using only cells in which both genes were detected are marked in the scatter plots. **C.** The workflow of the scLink method. In the first step, scLink calculates a robust co-expression matrix from the gene expression data. In the second step, scLink identifies a sparse gene network from the co-expression matrix using a penalized and data-adaptive likelihood approach.

To improve the construction of gene co-expression networks for single cells, we propose the scLink method to calculate the correlation between gene pairs, and then use a penalized and data-adaptive likelihood method to learn sparse dependencies between genes and construct sparse gene co-expression networks. One motivation of scLink is that the conventional Pearson and Spearman’s correlation coefficients do not provide an efficient approach to representing and interpreting gene associations given the high sparsity of single-cell gene expression data. For instance, we calculated the Pearson and Spearman’s correlation for a gene expression dataset of 109 immune B cells [12] (Figure S1), after normalization and log transformation as described in the next subsection. We only used 410 genes with at least a 10% detection rate, and the proportion of zero counts in the expression matrix is 71.2%. As an example, there are 886 gene pairs with a Pearson correlation in the range of [−0.15, −0.14], and 1302 gene pairs with a Spearman’s correlation in [0.14, 0.15], but their association varies in a much larger range when we recalculated the correlation only using the cells in which both genes were detected (Figure 1A and B). To more accurately infer gene co-expression networks that can capture functional gene modules, scLink has two major steps (Figure 1C). The first step is to calculate a robust co-expression matrix from the gene expression data to accurately represent the co-expression relationships among the genes. The second step is to identify a sparse gene network from the co-expression matrix using a penalized and data-adaptive likelihood approach.

### A robust estimator for measuring gene co-expression strength

Accurate and robust estimation of gene co-expression strength is the key to reliable inference of gene co-expression networks. Since single-cell gene expression data contain a high proportion of zero and inaccurate low counts due to both technical and biological variabilities [28], [34], the conventional Pearson or Spearman’s correlation coefficients are often not reliable for single-cell gene expression data, especially for genes whose expression values are highly sparse (Figure 1). In our previous work, we proposed a statistical method, scImpute, to address the excess zeros in scRNA-seq data [28]. Based on scImpute’s idea to identify the highly likely outliers (gene expression values that are not accurately measured), we propose a robust estimator for gene co-expression strength and use it to improve the inference of sparse gene co-expression networks.

Suppose the scRNA-seq data of a certain cell type (determined using marker genes or computational tools) are summarized as a read count matrix, with rows representing n cells and columns representing p genes. We normalize the count matrix by the library size of each cell, such that all cells have M reads after normalization. Typical choices for M include the median library size or a predetermined constant (*e.g.*, $10^{5}$) [16]. Denoting the normalized matrix as C, we apply log_10_ transformation to the count matrix to prevent a few large observations from being extremely influential. The resulting matrix is denoted as Y, with $Y_{\mathrm{ij}}=\log_{10}\left(C_{\mathrm{ij}}+1.01\right)$ (i=1,2,⋯,n;j=1,2,⋯,p). The pseudo-count 0.01 is added to avoid infinite values in parameter estimation.

We denote the log-transformed gene expression matrix without the pseudo-count as X, where $X_{\mathrm{ij}}=\log_{10}\left(C_{\mathrm{ij}}+1\right)$ $\left(i=1,2,\cdots ,n;j=1,2,\cdots ,p\right).$ In conventional methods, pairwise correlation coefficients are calculated using X to obtain the sample correlation matrix, based on which gene networks are constructed. In the scLink method, however, we add a filtering step to identify the accurately measured read counts, and rely on these counts in network inference, by adapting a mixture model used in scImpute. Similar mixture models have been shown to effectively capture the bimodal characteristic of single-cell gene expression data [34], [35], [36]. Specifically, for gene j, we assume its expression level is a random variable $Y_{j}$ following a Gamma-Normal mixture distribution, with a density function

(1) $$f_{Y_{j}}\left(y\right)=\lambda_{j}Gamma\left(y;\alpha_{j},\beta_{j}\right)+\left(1-\lambda_{j}\right)Normal\left(y;\mu_{j},\sigma_{j}\right)$$

where $\lambda_{j}$ is gene j’s non-detection rate, $\alpha_{j}$ and $\beta_{j}$ are the shape and rate parameters in the Gamma distribution, respectively, and $\mu_{j}$ and $\sigma_{j}$ are the mean and standard deviation in the Normal distribution, respectively. The Gamma distribution models the observed gene expression when the sequencing experiments fail to accurately capture gene j’s transcripts, while the Normal distribution models the actual gene expression levels.

Given the Gamma-Normal mixture distribution, the log-likelihood of gene j’s expression levels across all cells can be calculated as $l\left(\lambda_{j},\alpha_{j},\beta_{j},\mu_{j},\sigma_{j}\right)=\sum_{i=1}^{n}\log f_{Y_{j}}\left(y_{\mathrm{ij}};\lambda_{j},\alpha_{j},\beta_{j},\mu_{j},\sigma_{j}\right).$ We designed an Expectation-Maximization algorithm to estimate the parameters in the model presented in Equation (1) by maximizing the log-likelihood, and these estimates are denoted as ${\hat{\lambda }}_{j}$, ${\hat{\alpha }}_{j}$, ${\hat{\beta }}_{j}$, ${\hat{\mu }}_{j}$, and ${\hat{\sigma }}_{j}$, respectively. We can then filter the gene expression values based on the non-detection probability of gene j in cell i, which is estimated as

(2) $$d_{\mathrm{ij}}=\frac{{\hat{\lambda }}_{j}Gamma\left(Y_{\mathrm{ij}};{\hat{\alpha }}_{j},{\hat{\beta }}_{j}\right)}{{\hat{\lambda }}_{j}Gamma\left(Y_{\mathrm{ij}};{\hat{\alpha }}_{j},{\hat{\beta }}_{j}\right)+\left(1-{\hat{\lambda }}_{j}\right)Normal\left(Y_{\mathrm{ij}};{\hat{\mu }}_{j},{\hat{\sigma }}_{j}\right)}$$

Since $d_{\mathrm{ij}}\in \left(0,1\right)$ and a smaller $d_{\mathrm{ij}}$ indicates greater confidence on the observed gene expression $Y_{\mathrm{ij}}$, we can filter expression values by selecting a threshold t. Gene expression values with $d_{\mathrm{ij}}<t$ are considered to be accurately measured with high confidence, while expression values with $d_{\mathrm{ij}}\geq t$ are treated as missing values. We set t=0.5 in our analysis, as we have previously demonstrated that the selection of this threshold only impacts a tiny proportion of genes [28].

Given the identified accurate expression values and missing values, our robust estimator for measuring gene co-expression strength is defined as the pairwise-complete Pearson correlation coefficient. scLink then calculates the co-expression strength from gene expression matrix X, where $X_{\mathrm{ij}}=\log_{10}\left(C_{\mathrm{ij}}+1\right)$ (i=1,2,⋯,n;j=1,2,⋯,p). For genes $j_{1}$ and $j_{2}$, their robust correlation is calculated as

(3) $$r_{j_{1}j_{2}}=\frac{\sum_{i=1}^{n}\left(X_{ij_{1}}-{\overset{-}{X}}_{{∙j}_{1}}\right)\left(X_{ij_{2}}-{\overset{-}{X}}_{{∙j}_{2}}\right)I\left\{d_{ij_{1}}<t\right\}I\left\{d_{ij_{2}}<t\right\}}{\sqrt{a_{j_{1}j_{2}}}\sqrt{b_{j_{1}j_{2}}}}$$

where ${\overset{-}{X}}_{{∙j}_{1}}=\frac{1}{n}\sum_{i=1}^{n}X_{ij_{1}}$, ${\overset{-}{X}}_{{∙j}_{2}}=\frac{1}{n}\sum_{i=1}^{n}X_{ij_{2}}$, and

(4) $$a_{j_{1}j_{2}}=\sum_{i=1}^{n}{\left(X_{ij_{1}}-{\overset{-}{X}}_{{∙j}_{1}}\right)}^{2}I\left\{d_{ij_{1}}<t\right\}I\left\{d_{ij_{2}}<t\right\}$$

(5) $$b_{j_{1}j_{2}}=\sum_{i=1}^{n}{\left(X_{ij_{2}}-{\overset{-}{X}}_{{∙j}_{2}}\right)}^{2}I\left\{d_{ij_{2}}<t\right\}I\left\{d_{ij_{2}}<t\right\}$$

The pairwise robust correlation coefficients are used by scLink to construct gene co-expression networks as described in the next subsection. To improve the robustness of scLink, if the sample size for calculating correlation between genes $j_{1}$ and $j_{2}$ ($\sum_{i=1}^{n}I\left\{d_{ij_{1}}<t\right\}I\left\{d_{ij_{2}}<t\right\}$) is smaller than 10, we instead use the Pearson correlation coefficient for this pair of genes.

### The scLink method for gene network inference

To construct sparse gene co-expression networks from single-cell gene expression data, our scLink method adapts the Gaussian graphical model [29] and the penalized likelihood method [33], [37], which uses the principle of parsimony to select the simplest graphical model that adequately explains the expression data. We assume the actual gene expression values in each cell, without missing values being present due to technology limitations, to be a p-dimensional random vector $Z={\left(Z_{1},\cdots ,Z_{p}\right)}^{T}$ following a multivariate distribution $N\left(\mu ,\Sigma \right)$. Note that Z denotes the actual gene expression on the log_10_ scale and is a hidden variable not directly observable. We wish to estimate the concentration matrix $\Theta =\Sigma^{-1}$, where a zero entry $\theta_{j_{1}j_{2}}=0$ indicates the conditional independence between the two genes $j_{1}$ and $j_{2}$ given all other genes. In other words, if we consider an undirected graph G=(V,E), where V contains p vertices corresponding to the p genes and the edges are denoted as $E={\left\{e_{j_{1}j_{2}}\right\}}_{1\leq j_{1}\leq j_{2}\leq p}$. The edge between genes $j_{1}$ and $j_{2}$ is absent if and only if $\theta_{j_{1}j_{2}}=0$.

Given a random sample (n cells) of Z, a commonly used lasso-type estimator [33] takes the form

(6) $$\hat{\Theta }=\underset{\Theta ≻0}{\mathrm{argmax}}\log\det\left(\Theta \right)-tr\left(S\Theta \right)-\lambda {\left\|\Theta \right\|}_{1}$$

where $\log\det\left(\Theta \right)-tr\left(S\Theta \right)$ is proportional to the log-likelihood of Θ (ignoring a constant not depending on Θ) [37] and $\lambda {\left\|\Theta \right\|}_{1}=\lambda \sum_{j_{1}\neq j_{2}}^{p}\left|\theta_{j_{1}j_{2}}\right|\left(\lambda >0\right)$ is a penalty term adding a constraint on the number of non-zero elements in the concentration matrix. S denotes the estimated covariance matrix.

Recall that we summarize the observed gene expression matrix as X, where $X_{\mathrm{ij}}=\log_{10}\left(C_{\mathrm{ij}}+1\right)$ $\left(i=1,2,\cdots ,n;j=1,2,\cdots ,p\right).$ If we directly consider each matrix column $X_{∙1},\cdots ,X_{∙j}$ as a realization of Z, the covariance matrix could be estimated using the sample covariance matrix. However, due to limited detection capacity in scRNA-seq technologies as we have discussed above, the observed gene expression vectors ($X_{∙1},\cdots ,X_{∙j}$) cannot be directly treated as a sample of Z, and sample covariance matrix is not an ideal estimator of the covariance matrix Σ. In scLink, we estimate Σ using a robust estimator S, which is constructed with the robust correlation estimator introduced in the previous subsection. The elements in S are calculated as

(7) $$S_{j_{1}j_{2}}={\hat{\sigma }}_{j_{1}}{\hat{\sigma }}_{j_{2}}r_{j_{1}j_{2}}$$

where $r_{j_{1}j_{2}}$ is the robust correlation in Equation (3), and ${\hat{\sigma }}_{j_{1}}$ and ${\hat{\sigma }}_{j_{2}}$ are respectively the estimated standard deviation of genes $j_{1}$ and $j_{2}$ from the model presented in Equation (1). This idea of robust covariance estimation is motivated by a general framework for robust covariance calculation of high-dimensional data [38], and implemented with careful consideration of single-cell data characteristics.

In addition to proposing the robust estimator of covariance matrix, another feature of scLink is the formulation of a data-adaptive penalty term. We expect the penalty to be stronger on $\theta_{j_{1}j_{2}}$ if the robust correlation between genes $j_{1}$ and $j_{2}$ is weaker, and vice versa. Therefore, we propose a weighted penalty term $\lambda \sum_{j_{1}\neq j_{2}}^{p}\left(1-\right|r_{j_{1}j_{2}}\left|\right)\left|\theta_{j_{1}j_{2}}\right|$ (λ>0) to incorporate gene pair-specific information when adding the sparsity constraint.

In summary, the scLink estimator of the concentration matrix takes the form

(8) $${\hat{\Theta }}_{scLink}=\underset{\Theta ≻0}{\mathrm{argmax}}\log\det\left(\Theta \right)-tr\left(S_{p}\Theta \right)-\lambda \sum_{j_{1}\neq j_{2}}^{p}\left(1-\right|r_{j_{1}j_{2}}\left|\right)\left|\theta_{j_{1}j_{2}}\right|$$

where λ>0 and $S_{p}$ is a positive semidefinite approximation of S. In detail, $S_{p}=S+\left|\min\left\{0,\tau \right\}\right|I$, where τ is the smallest eigen value of S and I is an identity matrix [38]. There are multiple algorithms that can be implemented to solve the model presented in Equation (8), and scLink uses the QUIC algorithm [39] since its computational cost is O(p) and has a superlinear convergence rate. After we obtain the estimated $\hat{\Theta }$, it follows that ${\hat{E}}_{j_{1}j_{2}}=1$ if ${\hat{\theta }}_{j_{1}j_{2}}\neq 0$ and ${\hat{E}}_{j_{1}j_{2}}=0$ if ${\hat{\theta }}_{j_{1}j_{2}}=0$.

### Selection of the regularization parameter

In the model presented in Equation (8), the value of the regularization parameter λ would influence the sparsity level of the estimated concentration matrix and therefore the constructed gene network. Here we discuss two approaches that can be used to guide the selection of λ. The first approach is based on the Bayesian information criterion (BIC) [40]. For a particular value of λ, the BIC is calculated as

(9) $$BIC\left(\lambda \right)=ntr\left(S_{p}\hat{\Theta }\right)-n\log\det\left(\hat{\Theta }\right)+m\log n$$

where m is the total number of edges in the gene co-expression network. We can apply the model presented in Equation (8) on single-cell gene expression data with a sequence of regularization parameters, and select the value of λ that leads to the smallest BIC value. The second approach is to directly select λ based on the sparsity level of the constructed gene network. Suppose there is prior knowledge (*e.g.*, biological network databases) on the sparsity level, we can select the parameter that achieves the expected sparsity level of the gene co-expression network.

### Simulation of synthetic gene networks and expression data

We adapted the procedures in Mestres et al. [41] to simulate network structures. In each simulation setting, we first generated a block diagonal connectivity matrix $E_{p\times p}$, where each block had a hub-based or power-law topology, and the whole matrix also contained a fixed number of random connections between blocks. In the connectivity matrix, ${|E}_{j_{1}j_{2}}|=1$ if there was an edge between genes $j_{1}$ and $j_{2}$, and $E_{j_{1}j_{2}}=0$ if there was no edge between the two genes. This process was assisted with the R package ldstatsHD v1.0.1 [41]. Given the connectivity matrix, a partial correlation matrix was simulated by the following procedure:

$$\Lambda =\left[\lambda_{j_{1}j_{2}}\right],\lambda_{j_{1}j_{2}}=\left\{\begin{array}{c} Unif\left(0.4,0.7\right)ifE_{j_{1}j_{2}}=1;\\ Unif\left(-0.7,-0.4\right)ifE_{j_{1}j_{2}}=-1;\\ 0ifE_{j_{1}j_{2}}=0. \end{array}\right)$$

In case that Λ was not positive definite, we applied the transformation $\Lambda_{p}=\Lambda +\left|\min\left\{0,\tau \right\}\right|I$, where τ was the smallest eigen value of Λ and I was the identity matrix. We then calculated the corresponding correlation matrix R, and used it together with the gene expression mean and standard deviation estimated from a real scRNA-seq dataset [42] to simulate the synthetic gene expression matrix $X^{0}$ from a multivariate Gaussian distribution. The estimation of the gene expression parameters followed the procedures described in the previous subsection (A robust estimator for measuring gene co-expression strength). Next, we introduced zero counts to the gene expression matrix to mimic the observed zeros. Since the possibility of observing zero counts for a gene is negatively correlated with this gene’s mean expression in real data [28], [34], we calculated a probability for each entry in the gene expression matrix: $p_{\mathrm{ij}}=\exp\left(-\rho {\left(X_{\mathrm{ij}}^{0}\right)}^{2}\right)$, where ρ was a parameter controlling the dependence between non-detection probability and gene expression. Then, $p_{\mathrm{ij}}$ was the probability of observing a zero count for gene j in cell i. A binary indicator was sampled for each entry: $I_{\mathrm{ij}}∼Bernoulli\left(p_{\mathrm{ij}}\right)$, with $I_{\mathrm{ij}}=1$ indicating that the corresponding entry would be replaced by 0. Therefore, the final gene expression matrix was defined as X, where $X_{\mathrm{ij}}=X_{\mathrm{ij}}^{0}I\left\{I_{\mathrm{ij}}=0\right\}$. Repeating the aforementioned procedures with different values of ρ, we could generate synthetic single-cell gene expression matrices with known network topologies and different levels of sparsity. In our study, we used four different values of ρ: 0.07, 0.10, 0.13, and 0.16.

### Calculation of the robustness score

We used scLink as an example to describe how the robustness score was calculated. The robustness of the other two gene network inference methods, PIDC and GENIE3, was calculated using the same approach. For a selected cell number ($n_{r}$) and gene number (p), by randomly sampling $n_{r}$ cells from a given cell type for L times (L=10 in our analysis), we obtained L gene adjacency networks by scLink: $E^{\left(l\right)}\left(l\in \left\{1,2,\cdots ,L\right\}\right)$, where $E_{j_{1}j_{2}}^{\left(l\right)}=1$ if the two genes $j_{1}$ and $j_{2}$ had an edge in the l-th gene co-expression network; otherwise, $E_{j_{1}j_{2}}^{\left(l\right)}=0$. To simplify the notation, we denoted $E^{s}=\sum_{l=1}^{L}E^{\left(l\right)}$. The robustness of scLink was then calculated as

(10) $$RS=\frac{\sum_{j_{1}=1}^{p-1}\sum_{j_{2}=j_{1}+1}^{p}\sum_{l=1}^{L}\left(l-1\right)I\left\{E_{j_{1}j_{2}}^{s}=l\right\}}{\left(L-1\right)\sum_{j_{1}=1}^{p-1}\sum_{j_{2}=j_{1}+1}^{p}I\left\{E_{j_{1}j_{2}}^{s}>0\right\}}$$

For example, RS = 1 if the L inferred adjacency networks were exactly the same; RS = 0 if the L gene networks did not have any overlap.

### Availability of scRNA-seq data

The *Tabula Muris* dataset [43] is available at https://tabula-muris.ds.czbiohub.org/. The gene expression dataset of immune cells from breast cancer patients is available at Gene Expression Omnibus (GEO: GSE114727; https://www.ncbi.nlm.nih.gov/geo/) [12]. The gene expression dataset of definitive endoderm (DE) differentiation is available at GEO (GEO: GSE75748; https://www.ncbi.nlm.nih.gov/geo/) [44].

## Results

### scLink demonstrates efficiency in simulation studies

Our motivations for using simulated scRNA-seq data based on synthetic networks are two-fold. First, since the actual gene networks underlying real single-cell gene expression data are unknown, synthetic networks provide ground truth for comparing computational methods in a systematic and unbiased manner. Second, using simulated data, we can evaluate the performance of gene network inference methods given diverse network architectures and experimental settings. These results can help us investigate the advantages of each method in different scenarios.

**Figure 2:**
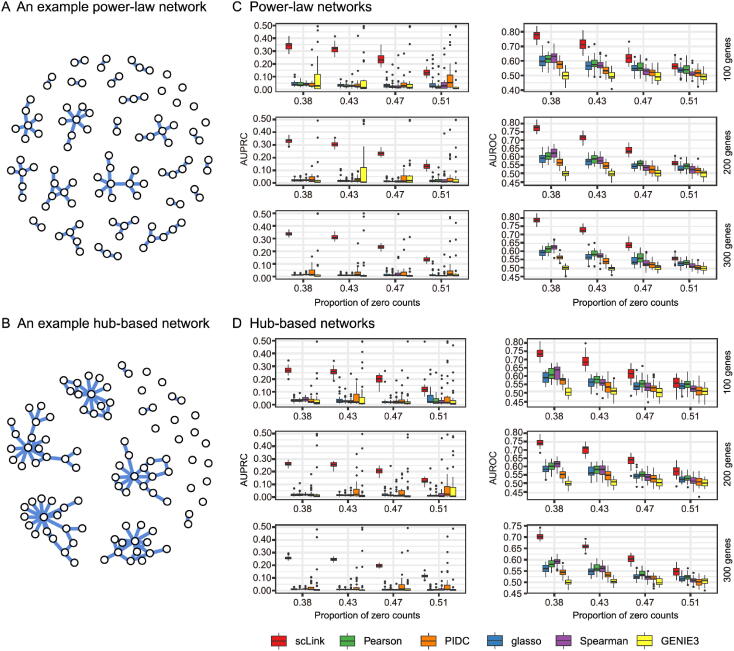
**Comparison of scLink and the other gene network inference methods on synthetic single-cell gene expression data. A.** An example 100-gene network with the power-law topology. **B.** An example 100-gene network with the hub-based topology. **C.** AUPRC and AUROC scores of scLink and the other five methods given gene expression data generated from the power-law networks. **D.** AUPRC and AUROC scores of scLink and the other five methods given gene expression data generated from the hub-based networks. The gene expression matrices have varying number of genes (100, 200, or 300) and proportion of zero counts. AUPRC, area under the precision–recall curve; AUROC, area under the receiver operating characteristic curve.

In our simulation, we considered two types of network topology: power-law networks and hub-based networks [41]. The power-law networks (Figure 2**A**) assume that the distribution of the node degrees (*i.e.*, the total number of edges of a node) follows a power law [45]. That is, $a\left(k\right)=k^{-\alpha }/\zeta \left(\alpha \right)$, where $a\left(k\right)$ denotes the fraction of nodes with degree k, α is a positive constant, and ζ(∙) is the Riemann zeta function. In contrast, in the hub-based networks (Figure 2B), a few nodes have a much higher degree than the rest nodes, and these high-degree nodes represent hub genes with critical functions in biological networks [17]. Using a carefully designed simulation framework (see Method), we generated synthetic single-cell gene expression matrices with known network topologies and different levels of sparsity. Therefore, we could evaluate the accuracy of a computationally inferred gene network by comparing it with the ground truth network.

We compared scLink with five alternative methods on the synthetic data to evaluate their accuracy in constructing gene co-expression networks. Among these five methods, PIDC infers genes’ dependencies in single cells based on the multivariate information theory [21]. GENIE3 was first developed to infer regulatory networks from bulk expression data [22], and was recently applied to single-cell expression data [24]. These two methods were demonstrated to have leading performance on simulated and real scRNA-seq data in a recent comparison [15]. In addition, we included glasso, a generic statistical method for estimating sparse networks [33], and its variants have been used to address different challenges in gene network construction [30], [46]. Finally, we also considered gene networks constructed by thresholding the Pearson or Spearman’s correlation coefficients, as these are commonly used statistical measures for constructing gene co-expression networks [17].

Since the gene networks are expected to be sparse, we used area under the precision–recall curve (AUPRC) as the primary criterion and area under the receiver operating characteristic curve (AUROC) as the secondary criterion, to achieve a fair and comprehensive comparison of the methods. The accuracy of scLink and glasso was obtained by evaluating them with different values of the regularization parameter (see Method). The accuracy of PIDC and GENIE3 was obtained by thresholding the estimated edge weights at different values. The accuracy of Pearson and Spearman’s correlation-based networks was obtained by thresholding the absolute values of correlation coefficients. For each type of network topology, we simulated synthetic single-cell gene expression data with 100, 200, and 300 genes. By changing the simulation parameters, we generated gene expression data with different sparsity levels. In each parameter setting, the simulation was independently repeated 50 times.

Our comparison results showed that for both power-law and hub-based network topologies, scLink had the best AUPRC and AUROC scores, outperforming glasso, PIDC, GENIE3, and the two correlation measures (Figure 2C and D). scLink demonstrated higher accuracy because it explicitly models the zero or low counts in single-cell data, providing a more robust estimator for gene co-expression strength to be used in the network inference step. Evaluating the performance of glasso, we found that it had a similar or even slightly lower accuracy compared with directly thresholding the Pearson correlation coefficients. This suggests that the generic penalized Gaussian graphical model is not very efficient for single-cell data. However, by incorporating improved co-expression measures and adding data-adaptive penalties to the gene–gene edges, scLink largely improved the accuracy of the graphical model. We also observed that most methods, including scLink, had increasing accuracy on less sparse gene expression data. A primary reason is that these data provide a larger effective sample size for network inference and contain fewer noises that could lead to false discoveries. This result suggests that it could be advantageous to filter out lowly expressed genes before network inference for real single-cell gene expression data. In addition, given the same sparsity level in single-cell data, most methods tended to have better performance on the power-law networks than the hub-based networks. A possible reason is that when multiple genes are simultaneously interacting with the same hub gene, it is very challenging to precisely distinguish the direct dependencies from the indirect ones among these genes only using the gene expression data.

As a proof-of-concept study, we also compared two modified versions of glasso with scLink on the simulated data. The first method, glasso-r, refers to the glasso method based on scLink’s robust correlation measure. It is the same as scLink except that it uses a constant weight of 1 instead of the adaptive weights in the penalty term. In other words, the penalty term is replaced with $\lambda \sum_{j_{1}\neq j_{2}}^{p}\left|\theta_{j_{1}j_{2}}\right|$ in glasso-r. The second method, glasso-f, refers to the combination of glasso and a filtering procedure. It filters out cells with greater than 70% of zero counts before applying the glasso approach. This reflects the practice to filter out low-quality cells in real practice. Our results based on both power-law and hub-based networks showed that glasso-f did not effectively improve the network construction accuracy compared with glasso (Figure S2). In addition, scLink achieved higher AUPRC and slightly lower AUROC than glasso-r, suggesting the additional benefit of using adaptive penalty for constructing single-cell gene networks. Furthermore, as a control study, we also compared scLink with the other five methods on simulated data without introducing an extra level of sparsity (Figure S3). In this control study, the synthetic data were generated as described in Method, except that the step of introducing zero counts was skipped. As expected, all the methods, especially the two methods based on Pearson and Spearman’s correlation, had improved accuracy compared with the performance on sparse gene expression data. This study demonstrates the unique challenge presented by the high level of sparsity in single-cell gene expression data, and the need to develop specific methods accounting for these data characteristics in the modeling step.

### scLink identifies cell type-specific gene networks from the *Tabula Muris* data

To evaluate scLink’s performance on real single-cell data and demonstrate its application to construct cell type-specific gene networks, we applied scLink to gene expression data derived from Smart-seq2 RNA-seq libraries [47]. This dataset from the *Tabula Muris* database includes 53,760 cells of 20 different tissues from 8 mice [43], providing a valuable opportunity to perform the analysis in a cell type-specific manner. We applied scLink to gene expression datasets of 59 cell types (each with at least 100 cells), using the top 500 highly expressed genes in each dataset. The proportion of zero counts in the 59 gene expression matrices ranged between 1.0% and 40.5%, and had a mean of 12.4%. The regularization parameters in scLink were selected as the smallest value from {1, 0.95, … , 0.05} such that the resulting networks had no more than 5% edges (6237 edges). After constructing the cell type-specific gene co-expression networks, we summarized the gene degrees, number of network communities (identified by the Louvain algorithm [48]), and community sizes in Figure S4.

**Figure 3:**
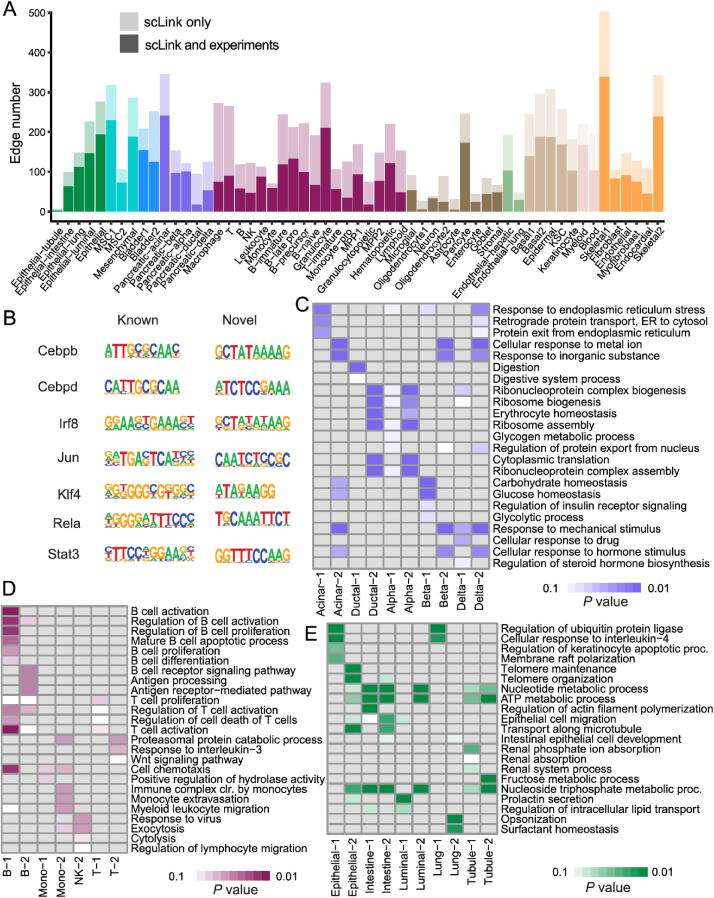
**Performance of scLink on the *Tabula Muris* dataset. A.** The numbers of TF–gene edges identified only by scLink and by both scLink and ChIP-seq experiments. **B.** Known and novel motifs of seven TFs identified from the promoter regions of genes connected to these TFs by scLink. **C.** Enriched GO terms in the two largest gene modules identified from pancreatic cell types. **D.** Enriched GO terms in the two largest gene modules identified from immune cell types. **E.** Enriched GO terms in the two largest gene modules identified from epithelial cell types. FDR-adjusted *P* values are shown in the heatmaps, and *P* values greater than 0.1 are shown in gray. TF, transcription factor; FDR, false discovery rate.

Since the true underlying gene networks were unknown for real gene expression data, we investigated the identified edges between genes and known TFs. For each cell type, we calculated the number of identified edges connected to known TFs in scLink’s results, and assessed their overlap with the TF–target edges discovered in previous ChIP-seq experiments [49], [50], [51]. Since the ChIP-seq experiments were performed using bulk data from human or mouse tissues instead of single cells, we pooled the TF–target pairs from the ChIP-seq experiments for the comparison, resulting in a database of 310 TFs. We used this database as a reference to investigate scLink’s performance, but we note that it’s not appropriate to treat this database as the ground truth. Our results showed that a substantial proportion of the identified TF–gene edges by scLink were previously discovered in ChIP-seq experiments (Figure 3**A**). This proportion ranged from 15.6% to 89.5% among different cell types with a median of 59.3%. Especially, scLink’s results had relatively high consistency with the ChIP-seq database in the epithelial, mesenchymal, pancreatic, epidermal, and muscle cell types, with a median overlapping proportion of 65.5%, 69.8%, 64.1%, 61.0%, and 65.4%, respectively (Figure 3A). As a comparison, we also applied five alternative network construction methods (described in the simulation studies) to the *Tabula Muris* data: Pearson correlation, Spearman’s correlation, PIDC, glasso-f, and glasso-r (Table S1). For these methods, the median proportions of identified TF–gene edges that were previously discovered in ChIP-seq experiments were 53.0%, 53.6%, 63.5%, 56.4%, and 58.1%, respectively. We found that scLink and PIDC generally lead to a higher consistency with the bulk tissue ChIP-seq database.

Since some TF–gene edges identified by scLink were not previously observed from ChIP-seq experiments, we performed a motif analysis using HOMER [52] to study if the genes connected to the same TF by scLink shared common motifs in their promoter regions. Our motif analysis identified both known and novel motifs for a group of TFs, including Cebpb, Cebpd, Irf8, Jun, Klf4, Rela, and Stat3 (Figure 3B). The motif analysis showed that genes connected to the same TF did have shared sequence features in their regulatory sequences and were likely to be co-expressed. In summary, the aforementioned analyses demonstrate that, by estimating the single-cell gene networks, scLink is able to identify edges between TFs and their potential target genes, even though scLink does not rely on any prior information of known TFs.

To further validate the biological functions of gene co-expression networks estimated by scLink, we investigated gene modules in the networks for the pancreatic, immune, and epithelial cell types. These modules were supposed to represent groups of highly co-expressed genes that shared similar biological functions or pathways in the corresponding cell types. For each cell type, we calculated the partial correlation matrix of the genes based on the estimated concentration matrix $\hat{\Theta }$ by scLink (see Method). Then, we performed hierarchical clustering using (1−|partial correlation|) as the distance measure. Next, we divided the genes into separate modules by cutting the dendrogram at a height of 0.85. Finally, we performed the Gene Ontology (GO) enrichment analysis on the gene modules.

The enriched GO terms in the two largest gene modules of each cell type are displayed in Figure 3C–E. For the pancreatic cells (Figure 3C), we found that GO terms related to protein transportation and digestive system process were enriched in gene modules of exocrine cells (acinar and ductal cells), while terms related to glycogen metabolic process, glucose homeostasis, and cellular response to hormone stimulus were enriched in gene modules of endocrine cells (alpha, beta, and delta cells). In addition, “regulation of insulin receptor signaling pathway” was only enriched in a gene module of beta cells, which has a critical role in insulin regulation. In contrast, “regulation of steroid hormone biosynthetic process” was only enriched in a gene module of delta cells, which secretes the hormone somatostatin. For the immune cells (Figure 3D), we found that GO terms related to B cell activation or proliferation were enriched in the largest gene module of B cells, and the terms related to B cell receptor signaling pathway and antigen processing were enriched in the second-largest module of B cells. In contrast, GO terms enriched in monocyte gene modules were related to monocyte extravasation and monocyte immune complex clearance. For the epithelial cells (Figure 3E), the enriched GO terms in gene modules also demonstrated cell type-specific biological functions.

**Figure 4:**
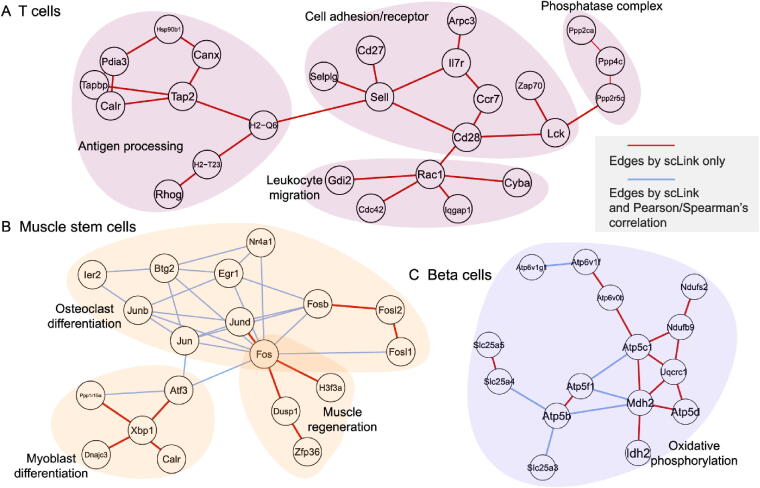
**Gene networks inferred by scLink overlap with functional protein interaction networks. A.** Edges inferred by scLink only or both scLink and correlation approaches for T cells. **B.** Edges inferred by scLink only or both scLink and correlation approaches for skeletal muscle satellite stem cells. **C.** Edges inferred by scLink only or both scLink and correlation approaches for pancreatic beta cells. Edges inferred only by scLink are displayed in red. Edges that were identified by both scLink and correlation approaches are displayed in blue. All the displayed edges are consistent with known protein interactions in the STRING database. Functional gene modules are grouped by the shaded area.

To investigate if the edges identified by scLink could improve the identification of molecular pathways and functional gene modules, we studied the inferred gene networks in T cells, skeletal muscle satellite stem cells, and pancreatic beta cells as three examples. In each scLink network, we focused on the largest connected component supported by known protein interactions in the STRING database [53]. We found that scLink could identify gene interactions that would be missed using a conventional approach with the Pearson or Spearman’s correlation (File S1). In T cells (Figure 4**A**), the inferred network by scLink contained four modules corresponding to gene sets of different functions, but these modules were not reported in correlation-based networks with the same sparsity level. Genes in the cell adhesion/receptor module are involved in the pathways of cell adhesion, cell surface interactions, and cell surface receptors; genes in the antigen processing module are responsible for antigen processing and presentation and immunoregulatory interactions [54]. In addition, the smallest module contained three genes associated with protein serine/threonine phosphatase complex, which has been shown to be a requisite of T cells’ functions [55]. In the muscle stem cells (Figure 4B), the inferred network contained a module of 11 genes playing key roles in osteoclast differentiation [56]. In this module, three edges were only identified by scLink. In addition, the network also contained a four-gene module involved in muscle regeneration and a five-gene module with roles in myoblast differentiation, both of which were also missed by the conventional correlation approach. In the pancreatic beta cells (Figure 4C), the inferred network identified a module of 15 genes responsible for oxidative phosphorylation, which plays an important role in beta cells’ proliferation, survival, and response to rising blood glucose. The aforementioned results demonstrate scLink’s ability to identify edges between genes with direct interactions or similar biological functions. We also investigated the largest connected components supported by known protein interactions in the gene networks based on Pearson or Spearman’s correlation, PIDC, glasso-f, and glasso-r (File S1). The largest gene modules in these networks were enriched with genes coding for ribosomal proteins (Table S2) and/or GO terms of biosynthetic and metabolic processes (Tables S3–S7). These results show that the gene modules identified by these methods capture less cell type-specific information and functional relevance than those gene modules identified by scLink.

### scLink identifies gene network changes in breast cancer

We next applied scLink to a single-cell dataset of breast cancer to study if scLink can help construct and compare gene co-expression networks in healthy and disease states. We downloaded the gene expression data of immune cells in the tumor (656 cells) and matched breast tissue (211 cells) from the same patient [12]. These data were obtained using the inDrop platform [57]. We separately applied scLink to data from the normal and tumor tissues, using the top 500 highly expressed genes in the normal tissue. The proportions of zero counts in the normal and tumor expression matrices were 49.0% and 66.0%, respectively. The regularization parameters in scLink were selected as the smallest value in {1.2, 1.1, … , 0.5} such that the inferred network had no more than 5% edges (6237 edges). In the identified gene network for the normal sample, the gene degree ranged from 2 to 103 with an average of 24.6. The 89 communities determined by the Louvain algorithm had an average size of 5.6. In the tumor sample, the gene degree ranged from 2 to 137 with an average of 26.2. The 63 communities determined by the Louvain algorithm had an average size of 7.9.

**Figure 5:**
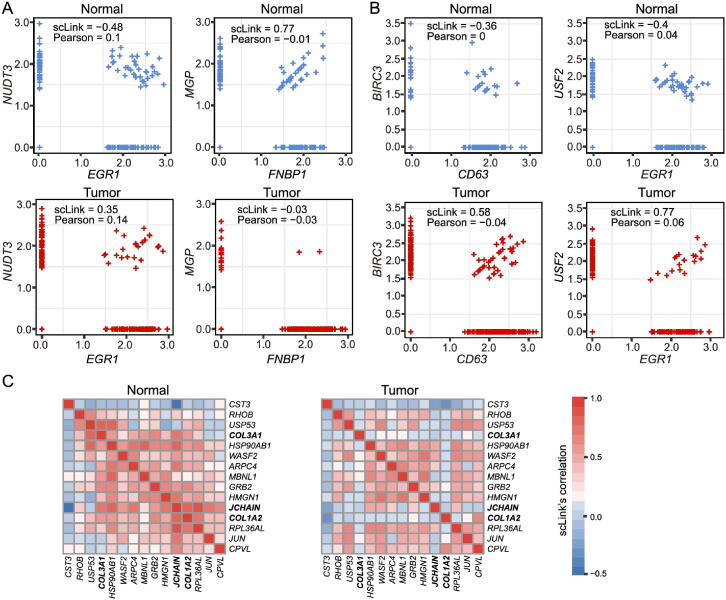
**scLink identifies differential co-expression relationships between normal and breast cancer tissues. A.** The log_10_-transformed expression of two gene pairs (*NUDT3* and *EGR1*; *FNBP1* and *MGP*) which were connected in the scLink network in immune cells of the normal tissue but not in the tumor tissue. Both scLink’s and Pearson correlation coefficients are displayed. **B.** The log_10_-transformed expression of two gene pairs (*CD63* and *BIRC3*; *EGR1* and *USF2*) which were connected in the scLink network in immune cells of the tumor tissue but not in the normal tissue. **C.** The correlation matrices (in the normal and tumor tissues) of a 15-gene module identified by scLink from the immune cells in the normal tissue. Genes showing opposite co-expression relationships with the other genes between the two conditions are highlighted in bold.

Comparing the two inferred gene co-expression networks, we found 453 differential edges with a greater than 0.5 change in scLink’s correlation and only present in the normal sample but not in the tumor sample (Figure S5A). We assessed the statistical significance of scLink’s correlation for the 453 edges using a bootstrap approach (File S1), and 84.5% edges had a P value < 0.05 after adjusting for the false discovery rate (FDR) (Figure S6A). For example, in the normal sample, *FNBP1* was co-expressed with *MGP*, whose down-regulation is associated with better survival in breast cancer [58] (scLink’s correlation = 0.77, adjusted P = 0) (Figure 5**A**). However, they were expressed in an independent manner in the tumor sample (scLink’s correlation = −0.03). As another example, scLink identified an edge between *EGR1* and *NUDT3* in the normal sample (scLink’s correlation =  − 0.48, adjusted P = 0.004) but not in the tumor sample (scLink’s correlation = 0.35), and both genes were reported to have regulatory roles in breast cancer (Figure 5A) [54], [59].

Meanwhile, we identified 1384 differential edges with a greater than 0.5 change in scLink’s correlation and only present in the tumor sample but not the normal sample (Figure S5B). We also assessed the statistical significance of scLink’s correlation for these edges in the tumor condition, and 90.0% edges had a FDR-adjusted P value < 0.05 (Figure S6B). For instance, *EGR1* and *USF2* were highly co-expressed in the tumor sample (scLink’s correlation = 0.77, *P* = 0.01) but not in the normal sample (Figure 5B). A similar expression pattern was observed between *CD63* and *BIRC3* (scLink’s correlation = 0.58, adjustedP = 0 in the tumor sample). In addition to *EGR1*, *CD63* and *BIRC3* were also found to be associated with breast cancer [60], [61], while *USF2*′s role in breast cancer has not been clearly investigated. The aforementioned results demonstrate that, by comparing co-expression changes between healthy and disease states, it is possible to 1) identify new genes that are associated with a specific disease; and 2) investigate how co-expression and co-regulation of genes impact cell functions [12]. In contrast, the aforementioned co-expression changes could not be captured by the Pearson correlation coefficients (Figure 5A and B). Actually, the Pearson correlation changed by no more than 0.2 for 97.0% of the edges between the normal and tumor conditions (Figure S5C), suggesting its insensitivity in identifying important co-expression changes in single cells. For a more systematic comparison, we constructed Pearson correlation networks with the same level of sparsity. We then investigated the biological functions of the 50 genes with the largest degree changes between the normal and breast cancer conditions, using the gene networks constructed by scLink and Pearson correlation. We found that the top enriched GO terms in the 50 genes identified by scLink were related to immune responses of myeloid cells, leukocytes, and neutrophils. In comparison, GO terms enriched in the 50 genes identified by Pearson correlation were related to more general protein regulation processes and humoral immune response (Table S8).

By comparing the tight gene modules in the normal and tumor samples, we could observe a dramatic change in the global network structure in addition to the change in individual edges. By performing hierarchical clustering using the partial correlation matrix estimated by scLink, we identified 12 modules with at least ten genes in the normal breast tissue (Figure S7). Similarly, we identified 11 modules with at least ten genes in the tumor tissue (Figure S8). However, the two sets of module assignments only had an adjusted Rand index of 0.10 and a normalized mutual information of 0.49, implying widespread rewiring of gene networks in breast cancer. For example, a 15-gene module identified from the normal samples was much less densely connected in the tumor sample (Figure S9). In this module, three genes (*COL3A1*, *COL1A2*, and *JCHAIN*) had opposite co-expression relationships with the other genes between the two conditions (Figure 5C), and these genes are involved in the regulation of immune response. Since the *COL3A1* and *COL1A2* genes are both in the pathway of scavenging by class A receptors, which are important regulators of immune responses to cancer [62], their co-expression changes in the tumor tissue may help us better understand the immune response processes in breast cancer.

### scLink identifies gene network changes from time course data

**Figure 6:**
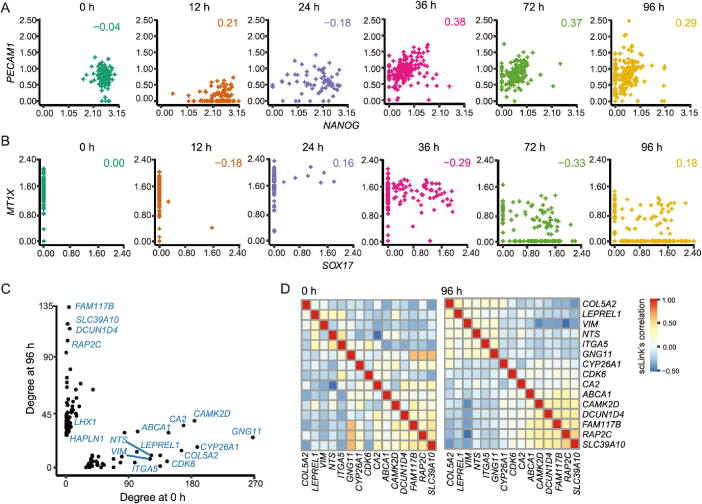
**scLink identifies gene network changes along the time course of definitive endoderm differentiation. A.** The log_10_-transformed expression of *PECAM1* and *NANOG* at different time points in the differentiation process of definitive endoderms. **B.** The log_10_-transformed expression of *MT1X* and *SOX17* at different time points in the differentiation process of definitive endoderms. Displayed numbers are correlation measures calculated by scLink. **C.** For genes whose degree changes are greater than 25 between 0 h and 96 h, their degrees at both time points are displayed. Labeled genes have degree changes greater than 100. **D.** scLink’s correlation matrices of genes with degree changes greater than 100 between 0 h and 96 h.

To further demonstrate scLink’s ability to quantify gene co-expression strength and infer gene networks in single cells under different conditions, we applied scLink to 758 single cells profiled by the Fluidigm C1 platform at 0 h, 12 h, 24 h, 36 h, 72 h, and 96 h of definitive endoderm (DE) differentiation [44]. We first compared scLink’s correlation between 51 lineage-specific marker genes [44] at different time points. Among the 1275 marker gene pairs, 240 pairs had a correlation change > 0.5 during DE differentiation (Figure S10A). For example, *NANOG* and *PECAM1* were weakly associated at the early time points (0 h, 12 h, and 24 h), but moderately associated at the late time points (36 h and 72 h) (Figure 6**A**); *MT1X* and *SOX17* did not demonstrate association at the early time points, but became negatively associated at 36 h and 72 h (Figure 6B). These findings are consistent with previous observations in DE differentiation studies [63], [64], but our results provide a detailed view of the association changes across the time points. These results may be used to interpret how these genes jointly regulate cell fate decisions in DE differentiation. In contrast, the Pearson correlation coefficients between these gene pairs were constantly low at almost all time points (Figure S10B), making it difficult to identify and interpret the genes’ association changes.

Next, we applied scLink to the gene expression levels from 0 h and 96 h data, using the top 1000 highly expressed genes in the whole dataset. The proportions of zero counts in the two expression matrices were 1.8% and 2.1%, respectively. The gene-level zero proportions were 0%–98.9% at 0 h and 0%–46.8% at 96 h. The regularization parameters were selected as the smallest value in {0.2, 0.19, … , 0.01} such that the inferred network had no more than 1% edges (4995 edges). In the 0 h gene network, the gene degree ranged from 2 to 274 with an average of 11.9, and the 188 communities identified by the Louvain algorithm had an average size of 5.3. In the 96 h gene network, the gene degree ranged from 2 to 136 with an average of 11.8, and the 296 communities identified by the Louvain algorithm had an average size of 3.4. By comparing the gene networks identified for the two time points, we found 595 differential edges whose corresponding gene pairs had a greater than 0.5 change in their co-expression (Figure S11).

To compare the differences in hub genes between the two time points, we assessed the change of gene degrees between the 0 h and 96 h networks. We found that genes with higher degrees at 0 h were enriched with GO terms relevant to mitosis, cell cycle, and chromosome separation, while genes with higher degrees at 96 h were enriched with GO terms relevant to regulation of cell differentiation and organismal development (Figure S12). Among genes with the largest degree changes, we observed three lineage-specific marker genes, *LHX1*, *HAPLN1*, and *GNG11* (Figure 6C). In addition, among genes with larger degrees at 0 h than at 96 h, we observed *CYP26A1*, *CDK6*, *VIM*, and *ITGA5* (Figure 6C), which have been shown to have regulatory roles in cell proliferation and/or cell differentiation [65], [66], [67]. In addition, we observed two tight gene modules at 96 h but only one such module at 0 h (Figure 6D), implying that joint expression of a gene set, including *COL5A2*, *LEPREL1*, *VIM*, *NTS*, *ITGA5*, and *GNG11,* may be critical to the differentiation of embryonic stem cells. The aforementioned results show that the gene co-expression networks identified by scLink from different time points provide important clues regarding transcriptional changes in the differentiation process of DE. In contrast, we also constructed Pearson correlation networks with the same level of sparsity, and investigated the biological functions of genes with high degrees at 0 h and 96 h. We found that 30.9% of the 1000 genes had the same direction of degree change in the scLink and Pearson correlation networks (Figure S13A). Unlike the scLink networks, genes with higher degrees at 0 h in the Pearson correlation network were enriched with GO terms of translation processes, while genes with higher degrees at 96 h were enriched with GO terms relevant to apoptosis (Figure S13).

### scLink demonstrates computational efficiency and robustness

To evaluate the computational efficiency and robustness of gene network construction, we compared the performance of scLink with PIDC and GENIE3 based on scRNA-seq data from two protocols, Smart-seq2 [47] and 10X Genomics [68]. For the Smart-seq2 protocol, we selected four cell types, late pro-B cells, bladder urothelial cells, myeloid cells, and microglial cells, from the *Tabula Muris* dataset. The cell numbers of the four cell types were 306, 684, 1208, and 4394, respectively. For each cell type, we selected 100, 200, and 500 highly expressed genes for network construction. In order to assess the robustness of the three methods given random variation, for each cell type, we randomly selected half of the cells for network construction and independently repeated the procedure ten times. The robustness score of each method was calculated based on the consistency between the ten inferred networks of the same cell type (see Method). For each method, the summarized computation time and memory usage were averaged across the ten repeated experiments. Our results showed that scLink achieved higher robustness than PIDC and GENIE3 while requiring much less computation time and memory usage (Figure S14). For the 10X Genomics protocol, we evaluated scLink and PIDC based on scRNA-seq data of 10,085B cells [68]. Since the computation time of GENIE3 for Smart-seq2 data exceeded 105 s (2.8 h) when 500 genes and 2197 cells were used, we did not test it on the large-scale 10X data. For the B cells, we selected the 500 and 1000 highly expressed genes for network construction. In order to assess the robustness, we randomly selected 5000 or 8000 B cells for network construction and independently repeated the procedure ten times. scLink again achieved higher computational efficiency and robustness than PIDC (Figure S15). In addition, scLink finished the computation in fewer than 100 s to construct a co-expression network of 1000 genes using 8000 cells. We also noticed that the best robustness score achieved by scLink was around 0.5, and this could be explained by two major reasons. First, the correlation calculation and network inference were inevitably affected by the random variation in single-cell gene expression data, leading to variation of identified edges for randomly sampled cells of the same cell type. Second, since the cell types in real scRNA-seq data were also computationally inferred, there might exist cell subtypes that had biologically different gene co-expression networks. The aforementioned experiments were performed using a Ubuntu 16.04.5 system and two 8-core CPUs of Intel Xeon CPU E5-2670 at 2.60 GHz.

## Discussion

In this work, we develop a method called scLink to improve the construction of sparse gene co-expression networks based on single-cell gene expression data. We first propose a new correlation measure for gene co-expression relationships to account for the sparsity feature of single-cell gene expression data. Next, relying on the more robust correlation measure, scLink identifies gene co-expression networks using a penalized and data-adaptive likelihood model. Our simulation studies show that scLink has the best accuracy in gene network construction compared with five other state-of-the-art methods, given different network topologies (hub-based or power-law), gene numbers, and sparsity levels of gene expression. Our results based on the *Tabula Muris* database show that scLink is able to identify cell type-specific networks and functional gene modules, and the edges inferred by scLink can capture regulatory relationships between gene pairs. Our real data studies also demonstrate scLink’s ability to help identify co-expression changes and gene network rewiring between healthy and disease states. In addition, scLink is also demonstrated to reveal network differences and critical hub genes in time course data, such as those from the DE differentiation process.

To demonstrate the applications of scLink and disseminate the research findings in our real data studies, we develop a web application of scLink (https://rutgersbiostat.shinyapps.io/sclink/). This application provides an interactive platform for users to subset and visualize the cell type-specific correlation matrices and gene networks constructed by scLink (Figure S16). For easy application of scLink to additional single-cell gene expression datasets, we also implement the methods in the R package scLink (https://github.com/Vivianstats/scLink).

In the simulated and real data studies, we construct gene networks with 100–1000 genes. In actual applications of scLink, we also suggest a gene filtering step based on the gene detection rates or the mean expression levels [15]. Instead of selecting an arbitrary number of genes to be retained, researchers can also set a threshold such that genes of particular interest will be included. The rationale for implementing this filtering step is that genes with small detection rates and expression levels often have low biological relevance and do not provide sufficient information for co-expression estimation. Including these genes might increase false edges in the gene networks. For example, our simulation studies demonstrate that the accuracy of network construction decreases with increasing level of sparsity in single-cell data, regardless of the method being used (Figure 2). Given the relatively high sparsity level of data generated by droplet-based scRNA-seq protocols [69], this filtering step is especially necessary on gene expression data from these protocols. When it is of interest to construct a network of thousands of genes, it is still possible to directly apply scLink, but the likelihood optimization step would be more time-consuming because it involves large-scale matrix operations. An alternative approach is to first divide the genes into a few major modules based on scLink’s correlation, and then separately apply scLink to each module to identify co-expression networks.

Since the first step of scLink is partially motivated by our scImpute method [28] and additional imputation methods for single-cell gene expression data have also become available, an alternative approach to constructing gene co-expression networks is to apply conventional network inference methods to imputed gene expression data. However, we would like to discuss two potential issues with this approach. First, previous studies have shown that imputed data may still be much sparser than bulk data, even though containing fewer zero counts than the observed single-cell data [28], [70]. Therefore, conventional network construction methods designed for bulk data may still have poor performance even when applied to imputed single-cell data. Second, imputation of gene expression could be a time-consuming step depending on the cell number of the data. By skipping the imputation step and directly accounting for the sparsity issue in co-expression calculation, scLink can achieve better computational efficiency.

Even though our simulation and real data studies demonstrate the great potential of scLink on different types of single-cell gene expression data, we need to interpret the results with caution, since the edges identified by scLink are based on statistical dependencies and do not have directions. These edges may capture the actual regulatory relationships, such as those between TFs and their target genes. However, the inferred edges may also represent co-regulatory relationships of genes regulated by common TFs. In addition, we may also identify edges between genes that are responsible for similar biological functions and demonstrate coordinated expression patterns. It is not feasible to directly distinguish the aforementioned different types of edges using only gene expression data, but scLink’s results provide good candidates for further computational and/or experimental validations. For example, single-cell ChIP-seq experiments could be designed and prioritized based on scLink’s identified TF–gene pairs [71]. It is also possible to take advantage of existing databases of TFs and protein–protein interactions at the validation step, but the knowledge derived from previous bulk tissue research does not necessarily reflect the true scenario in single cells [25].

As discussed in several recent methods, it is possible to more directly infer gene regulatory relationships instead of co-expression relationships if temporal information is available for the single cells. Some of these methods take pseudo-time orders estimated by computational methods [26], [72], while others assume that actual time course data are available [73]. In real practice, as most scRNA-seq experiments are not performed along a time course, only pseudo-time orders may be available for the majority of datasets. However, since pseudo-time orders are only point estimates of physical time orders, it is important to consider how to quantify pseudo-time uncertainty and propagate this into the construction of gene regulatory networks. Aside from temporal information, additional experimental data, such as ATAC-seq or ChIP-seq data, have also been shown to assist the inference of gene regulatory networks in bulk tissue studies [74]. As single-cell multi-omics technologies and data integration methods continue to emerge and evolve [75], it will become possible to modify and extend existing bulk-tissue methods for single-cell data. The penalized likelihood approach used in scLink can incorporate the aforementioned additional information with flexibility. For instance, we can extend the penalty terms in scLink to apply different levels of regularization on each gene pair based on the epigenetic or chromatin accessibility information. This extension will be particularly helpful to more accurately infer the co-expression patterns among genes with low-to-mediate expression levels. Another future direction is to construct differential gene networks between biological conditions from scRNA-seq data. In our real data studies, we used a straightforward approach to identify differential edges based on the differences in scLink’s correlation strength. However, it is possible to extend the likelihood model to directly identify differential networks using scRNA-seq data from both conditions, as previously done for bulk tissue RNA-seq data [32], [76]. With the ongoing efforts of single-cell atlases such as the Human Cell Atlas [77] to better define cell types, states, and lineages, it will also become possible to investigate how gene co-expression and interactions differ in related tissue and cell types. In summary, we expect scLink to be a useful tool for inferring functional gene networks from single-cell gene expression data, with the potential to incorporate other omics data types as single-cell technologies continue to develop.

## Data availability

The scLink R package is available at https://github.com/Vivianstats/scLink. The corresponding web application is accessible through https://rutgersbiostat.shinyapps.io/sclink/.

## Competing interests

The authors declare that they have no competing interests.

### CRediT authorship contribution statement

**Wei Vivian Li:** Conceptualization, Methodology, Formal analysis, Software, Writing – original draft. **Yanzeng Li:** Software, Writing – review & editing.

## Supplementary material

Supplementary File S1 **Supplementary methods**

Supplementary Figure S1 **Distribution of the Pearson and Spearman's correlation between the 410 genes with at least 10% detection rate in B cells**

Supplementary Figure S2 **Comparison of scLink and glasso-based methods on synthetic single-cell gene expression data A.** AUPRC and AUROC scores of scLink and the other three methods given gene expression data generated from the power-law networks. The gene expression matrices have varying number of genes (100, 200, or 300) and proportion of zero expression is marked on the x-axis. **B.** AUPRC and AUROC scores of scLink and the other three methods given gene expression data generated from the hub-based networks.

Supplementary Figure S3 **Comparison of scLink and the other methods in the control study. A.** AUPRC and AUROC scores of scLink and the other five methods given gene expression data generated from the power-law networks. The gene expression matrices have varying number of genes (100, 200, or 300). **B.** AUPRC and AUROC scores of scLink and the other three methods given gene expression data generated from the hub-based networks.

Supplementary Figure S4 **Network statistics of the gene co-expression networks identified by scLink on the Tabula Muris data A.** Degree of genes in the cell-type-specific gene networks. **B.** Number of communities (identified by Louvain algorithm) in the cell-type-specific gene networks. **C.** Sizes of communities (identified by Louvain algorithm) in the cell-type-specific gene networks.

Supplementary Figure S5 **Summary of differential edges identified in the breast cancer application A.** Number of differential edges only present in normal immune cells, categorized by the absolute value of scLink’s co-expression change between normal and tumor conditions. **B.** Number of edges only present in tumor cells, categorized by the absolute value of scLink's co-expression change between normal and tumor conditions. **C.** Distribution of co-expression changes between normal and tumor cells as measured by scLink's correlation or the Pearson correlation coefficients.

Supplementary Figure S6 **Assessing the statistical significance of scLink correlation A.** Bootstrap P values of the scLink correlation for the 453 differential edges only present in the normal sample. **B.** Bootstrap P values of the scLink correlation for the 1384 differential edges only present in the tumor sample.

Supplementary Figure S7 **scLink identifies gene modules in immune cells of normal sample** Hierarchical clustering of genes was performed using the (1-|partial correlation matrix|) as the distance matrix. Modules were assigned by cutting the hierarchical dendrogram at the 90% percentile of all partial correlation coefficients. Only modules with more than 10 genes are displayed. The heatmap value denotes whether scLink identifies an edge for each pair of genes.

Supplementary Figure S8 **scLink identifies gene modules in immune cells of tumor sample** Hierarchical clustering of genes was performed using the (1-|partial correlation matrix|) as the distance matrix. Modules were assigned by cutting the hierarchical dendrogram at the 90% percentile of all partial correlation coefficients. Only modules with more than 10 genes are displayed. The heatmap value denotes whether scLink identifies an edge for each pair of genes in the tumor cells.

Supplementary Figure S9 Gene network is rewired in tumor cells The correlation matrices of a 15-gene module identified by scLink from the normal sample are displayed in the heatmaps. Only the correlation measures for identified edges in the normal or tumor sample are shown. Otherwise, the corresponding entries are colored in gray.

Supplementary Figure S10 **Application of scLink on time-course scRNA-seq data A.** The correlation calculated by scLink for 240 gene pairs at different time points. These 240 gene pairs have a correlation change greater than 0.5 along the time course of definitive endoderm differentiation. **B.** Log10-transformed gene expression of *PECAM1* and *NANOG*, *MT1X* and *SOX17* at different time points in the differentiation process. Displayed numbers are the Pearson correlation coefficients.

Supplementary Figure S11 **The correlation by scLink of 595 differential edges between 0 h and 96 h** These 595 gene pairs only have edges in either the 0 h network or the 96 h network, and have a correlation change greater than 0.5 along the time course of endoderm differentiation.

Supplementary Figure S12 **GO enrichment analysis of scLink’s results on time-course scRNA-seq data A.** Top enriched GO terms in genes with higher degrees at 0 h than at 96 h. We used all the genes whose degrees at 0 h are at least 20 greater than their degrees at 96 h. **B.** Top enriched GO terms in genes with higher degrees at 96 h than at 0 h. We used all the genes whose degrees at 96 h are at least 20 greater than their degrees at 0 h.

Supplementary Figure S13 **GO enrichment analysis of Pearson correlation networks for time-course scRNA-seq data A.** Comparison of genes’ degree changes from 0 h to 96 h in scLink and Pearson correlation networks. **B.** Top enriched GO terms in genes with higher degrees at 0 h than at 96 h. We used all the genes whose degrees at 0 h are at least 20 greater than their degrees at 96 h. **C.** Top enriched GO terms in genes with higher degrees at 96 h than at 0 h. We used all the genes whose degrees at 96 h are at least 20 greater than their degrees at 0 h.

Supplementary Figure S14 **Evaluation of computational performance based on Smart-Seq2 data A.** Computation time summarized in seconds. **B.** Memory usage summarized in megabytes. C. Robustness score.

Supplementary Figure S15 **Evaluation of computational performance based on 10x Genomics data A.** Computation time summarized in seconds. **B.** Memory usage summarized in megabytes. C. Robustness score.

Supplementary Figure S16 **The scLink web page provides an interactive application to demonstrate the usage and results of scLink**

Supplementary Table S1 **Proportions of the computationally identified TF-gene edges that were previously discovered in ChIP-seq experiments**

Supplementary Table S2 **Network analysis of the Tabula Muris data using correlation-based, PIDC, and glasso-based methods**

Supplementary Table S3 **GO terms enriched in the largest connected components of Pearson correlation-based networks constructed from Tabula Muris data**

Supplementary Table S4 **GO terms enriched in the largest connected components of Spearman’s correlation-based networks constructed from Tabula Muris data**

Supplementary Table S5 **GO terms enriched in the largest connected components of PIDC networks constructed from Tabula Muris data**

Supplementary Table S6 **GO terms enriched in the largest connected components of glasso-f networks constructed from Tabula Muris data**

Supplementary Table S7 **GO terms enriched in the largest connected components of glasso-r networks constructed from Tabula Muris data**

Supplementary Table S8 **GO enrichment analysis on genes with large degree changes between normal and breast cancer conditions**
