# Supplementary material for: scLink: Inferring Sparse Gene Co-expression Networks from Single-cell Expression Data
Source: Genomics Proteomics Bioinformatics. 2021 Jul 10;19(3):475–92. doi: 10.1016/j.gpb.2020.11.006 (PMC8896229; doi:10.1016/j.gpb.2020.11.006)
Supplement: Supplementary File S1 — Supplementary methods [file mmc1.pdf]

Supplementary Methods

Software information

The scLink method was implemented with the R package `scLink` v0.0.9. The R package `QUIC` v1.1 was used for estimation of concentration matrix in scLink. The Julia package NetworkInference v0.1.0 was used with default parameters to obtain PIDC's estimated edge weights. The R package `GENIE3` v1.4.3 was used with default parameters to obtain GENIE3's estimated edge weights. The motif analysis was performed using `HOMER` v4.4 with the default parameters except the following: "-start -500 -end 100 -len 8,10". The GO enrichment analysis was performed using the R package `clusterProfile` v3.10.1.

Network analysis of the Tabula Muris data

For single cell gene expression data of T cells, skeletal muscle satellite stem cells, and pancreatic beta cells, we applied scLink, the conventional correlation approach, PIDC, glasso-r, and glasso-f to infer the gene networks. The scLink method was applied as described in Methods and Results. For the correlation approach, we calculated the Pearson and Spearman's correlation matrices for each cell type, and only kept the top 5% edges with the largest absolute correlation coefficients in each network. For the PIDC method, we applied it to gene expression data of each cell type, and only kept the 5% edges with the largest estimated edge weights. For the glasso-r and glasso-f methods, we selected the regularization parameters (λ) as the smallest value from $\{1, 0.95, ..., 0.05\}$ such that the resulting network has no more than 5% edges. Therefore, the gene networks inferred from different approaches have the same level of sparsity and should be comparable.

Next, for each cell type, we obtained a sub-network from scLink's results by only keeping the edges with a confidence score greater than 0.8 in the STRING database. The confidence score is between 0 and 1, and a larger value represents a protein-protein interaction with overall higher confidence. We studied the largest connected component from the sub-network, and divided the edges into two categories in Figure 4: the edges only identified by scLink (red) and the edges identified by both scLink and the correlation approach (blue).

Similarly, for each network constructed using the other methods(Pearson or Spearman's correlation, PIDC, glasso-r, and glasso-f), we also obtained a sub-network by only keeping the edges with a confidence score greater than 0.8 in the STRING database. We then studied the largest connected component from the sub-network by summarizing the number of genes, the number of ribosomal protein genes, the proportion of gene-gene edges that is shared with scLink's results, and the enriched GO terms in the gene module.

Assessing the significance of scLink correlation

We propose an approach based on bootstrap to assess the statistical significance of scLink's correlation measure for gene co-expression strength. It has the following major steps:

1. Obtain the log-transformed and normalized gene expression matrix $\boldsymbol{X}_{n\times p}$, as described in Methods.

2. For genes j_1 and j_2,, calculate the robust scLink correlation r_{j_1,j_2} based on $\boldsymbol{X}$, as described in Methods.

3. In bootstrap iteration b, randomly sample n cells with replacement from the observed n cells to construct gene expression matrix $\boldsymbol{X}^{(b)}_{n\times p}$.

4. For genes j_1 and j_2, calculate the robust scLink correlation $r^{(b)}_{j_1,j_2}$ based on $\boldsymbol{X}^{(b)}$, as described in Methods.

5. Repeat steps 3 and 4 for $b = 1, \ldots, B$ (e.g., B = 500).

6. Calculate the one-sided p values as

$$p = \begin{cases} \sum_{b=1}^{B} \mathbb{1}\{r^{(b)}_{j_1,j_2} \leq 0\} & \text{if } r_{j_1,j_2} > 0 \\ \sum_{b=1}^{B} \mathbb{1}\{r^{(b)}_{j_1,j_2} \geq 0\} & \text{if } r_{j_1,j_2} < 0 \end{cases}$$

7. Adjust the p values based on the false discovery rate.
