# Supplementary figures and images for: scLink: Inferring Sparse Gene Co-expression Networks from Single-cell Expression Data
Source: Genomics Proteomics Bioinformatics. 2021 Jul 10;19(3):475–92. doi: 10.1016/j.gpb.2020.11.006 (PMC8896229; doi:10.1016/j.gpb.2020.11.006)

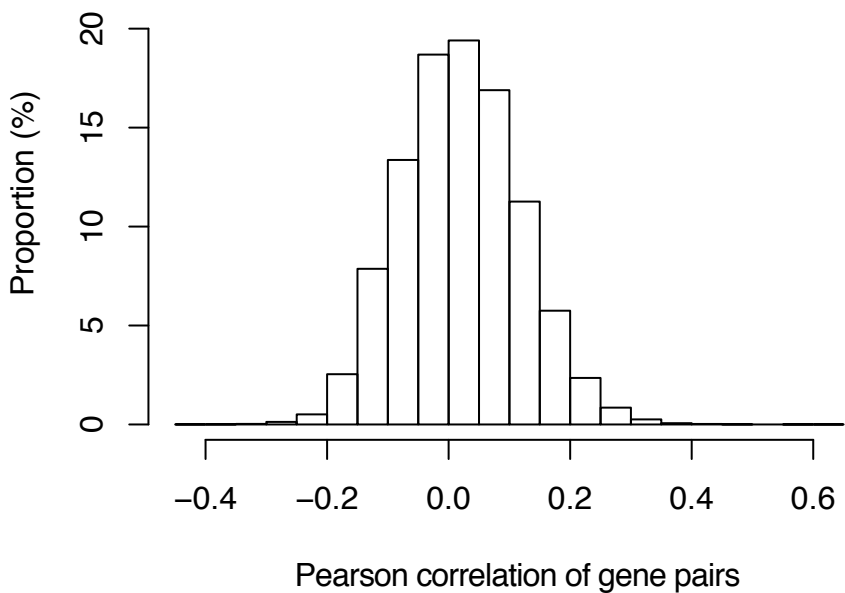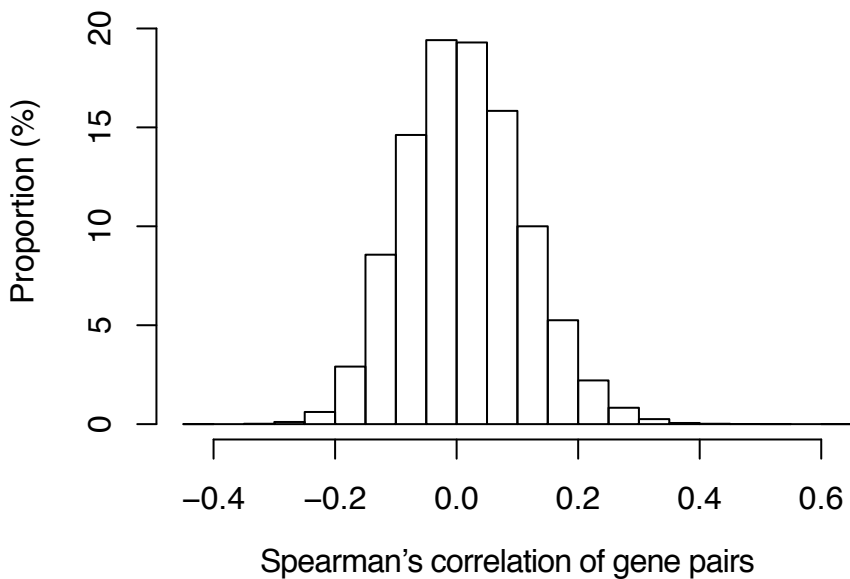

Supplement: Supplementary Figure S1 — Distribution of the Pearson and Spearman's correlation between the 410 genes with at least 10% detection rate in B cells [file mmc2.pdf]

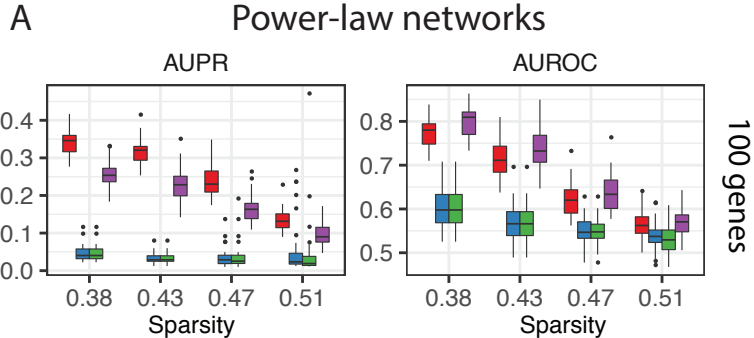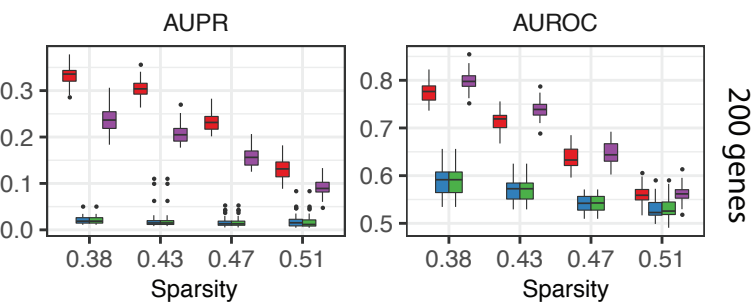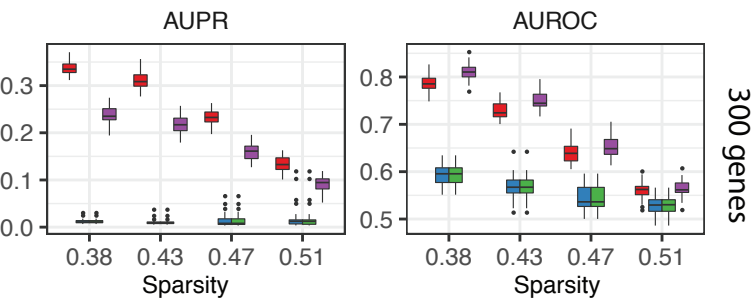

scLink glasso glasso-f glasso-r

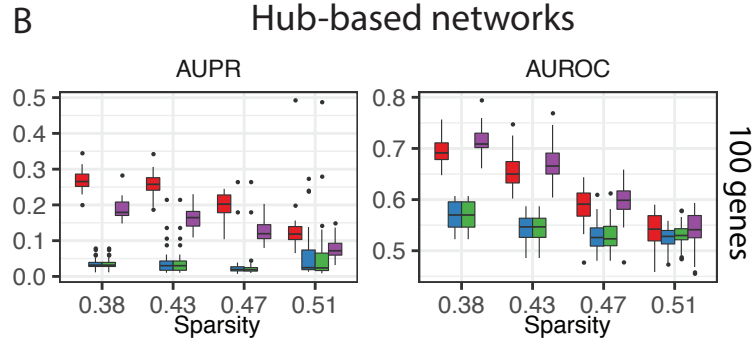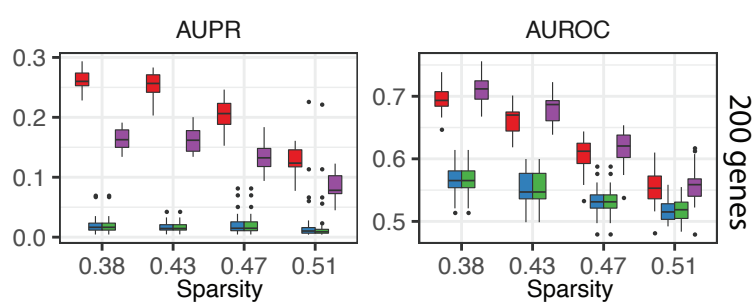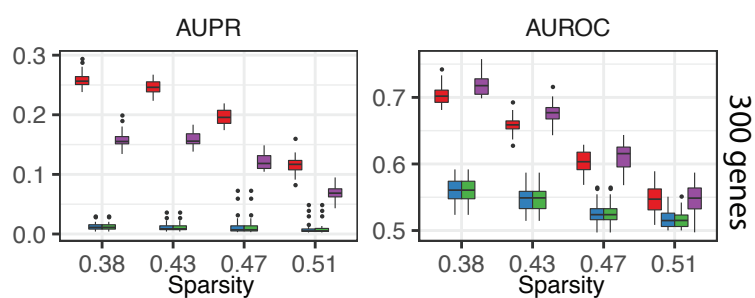

Supplement: Supplementary Figure S2 — Comparison of scLink and glasso-based methods on synthetic single-cell gene expression data A. AUPRC and AUROC scores of scLink and the other three methods given gene expression data generated from the power-law networks. The gene expression matrices have varying number of genes (100, 200, or 300) and proportion of zero expression is marked on the x-axis. B. AUPRC and AUROC scores of scLink and the other three methods given gene expression data generated from the hub-based networks. [file mmc3.pdf]

## A Power-law networks

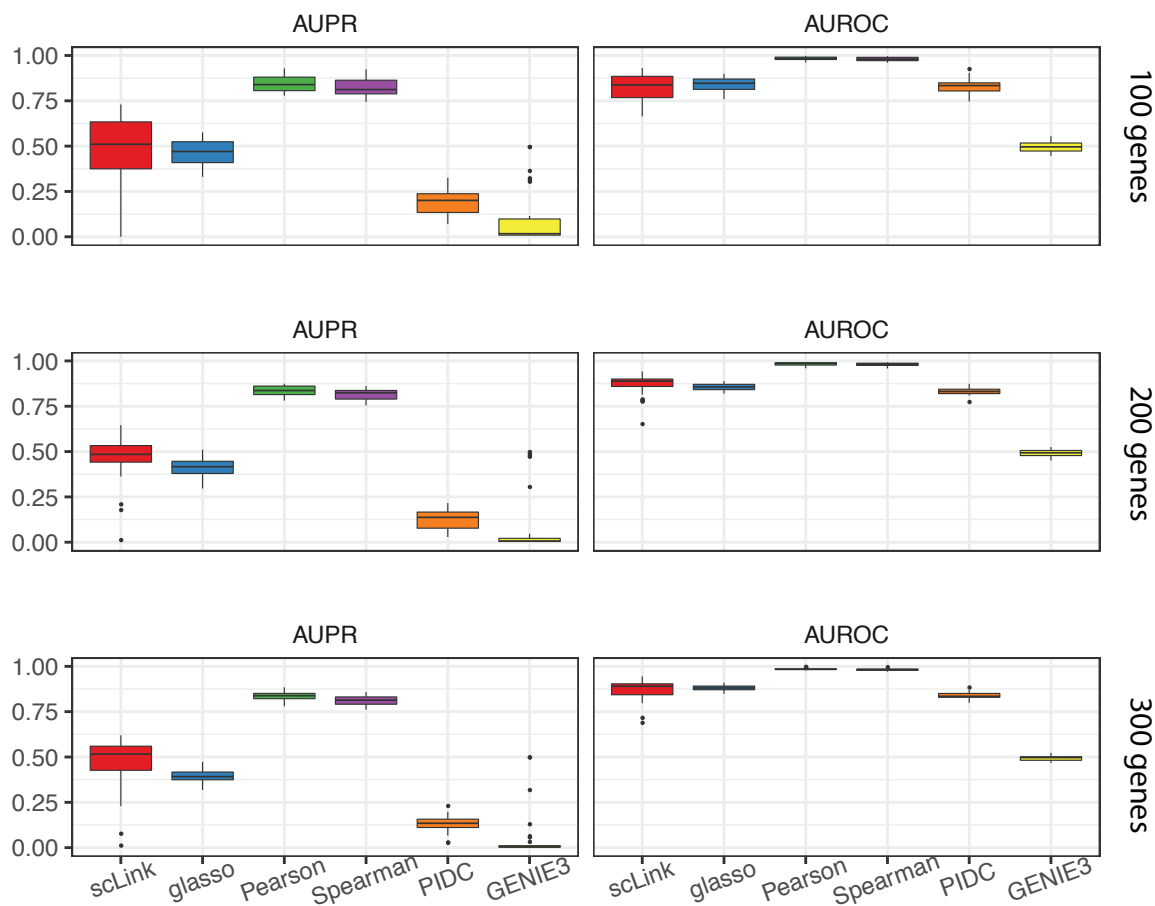

## B Hub-based networks

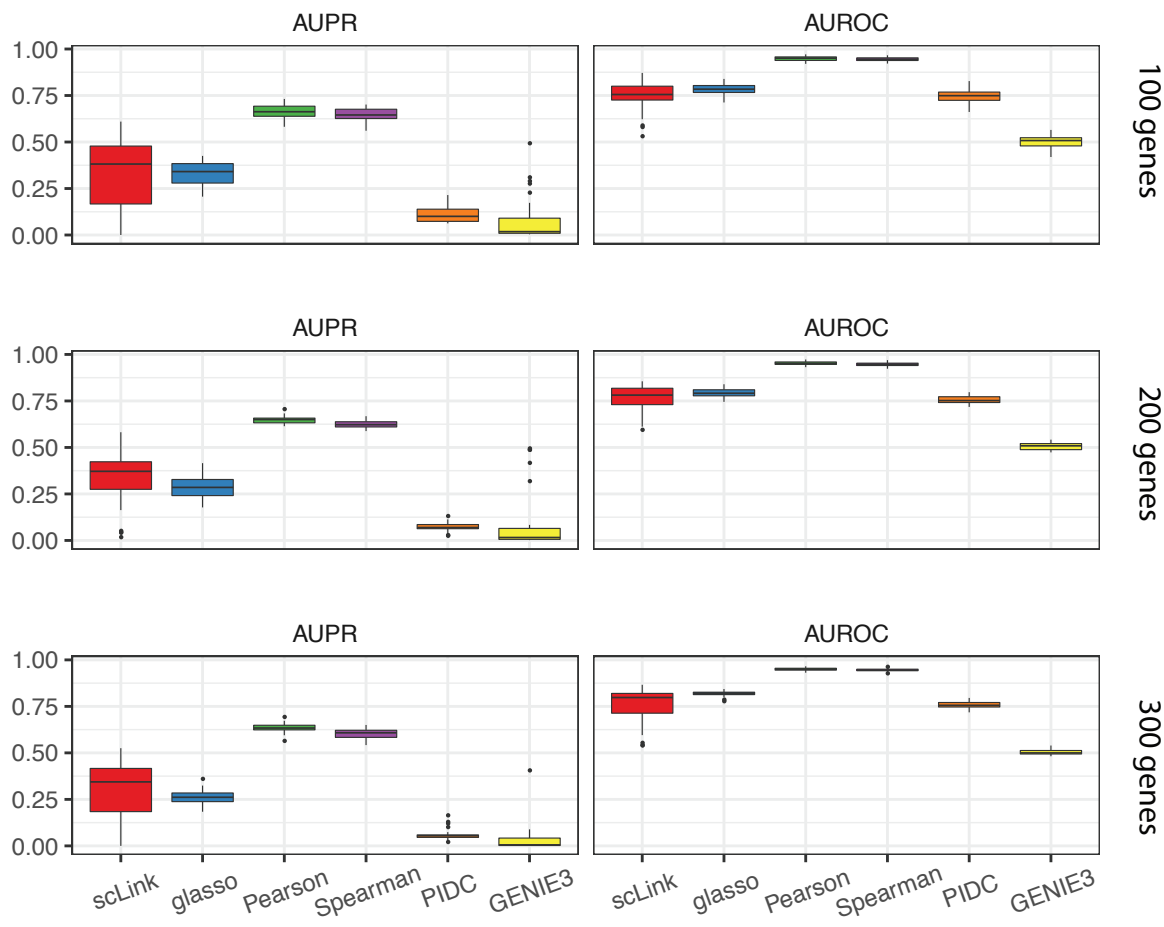

Supplement: Supplementary Figure S3 — Comparison of scLink and the other methods in the control study. A. AUPRC and AUROC scores of scLink and the other five methods given gene expression data generated from the power-law networks. The gene expression matrices have varying number of genes (100, 200, or 300). B. AUPRC and AUROC scores of scLink and the other three methods given gene expression data generated from the hub-based networks. [file mmc4.pdf]

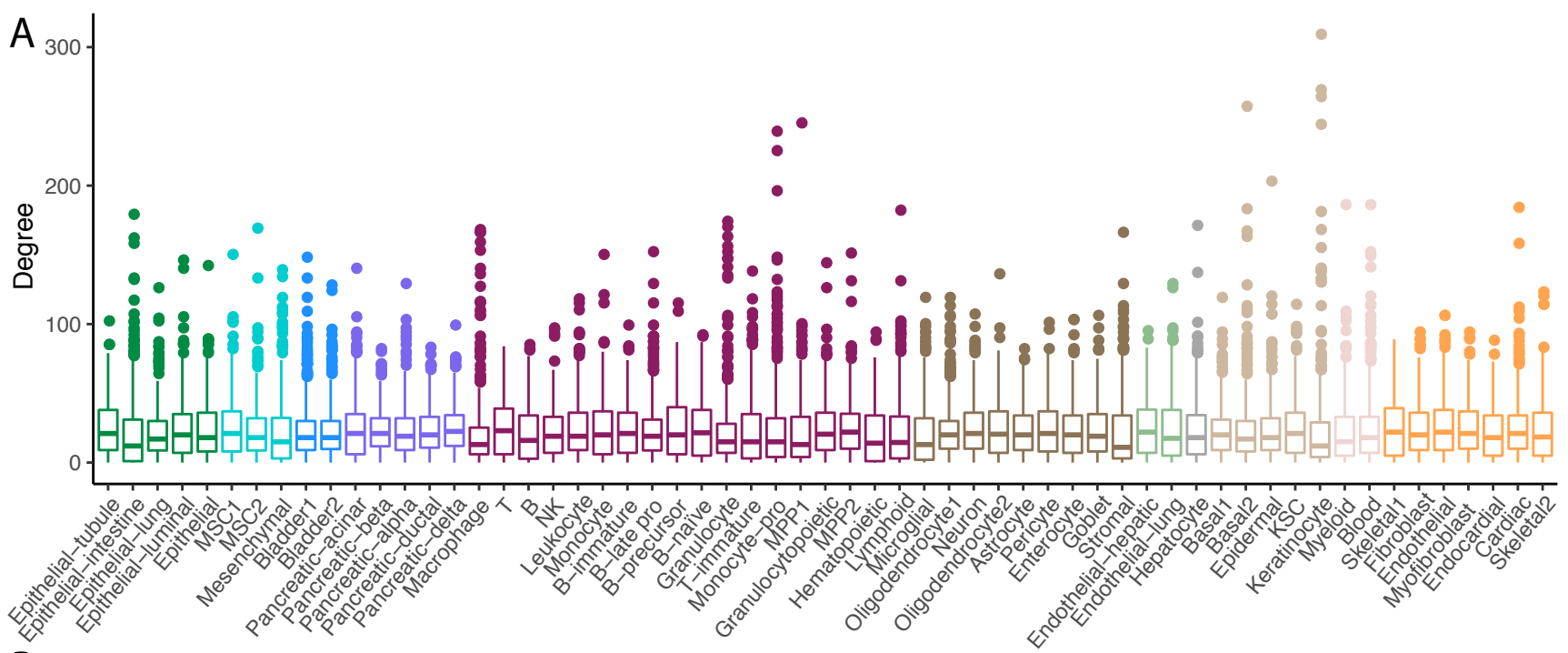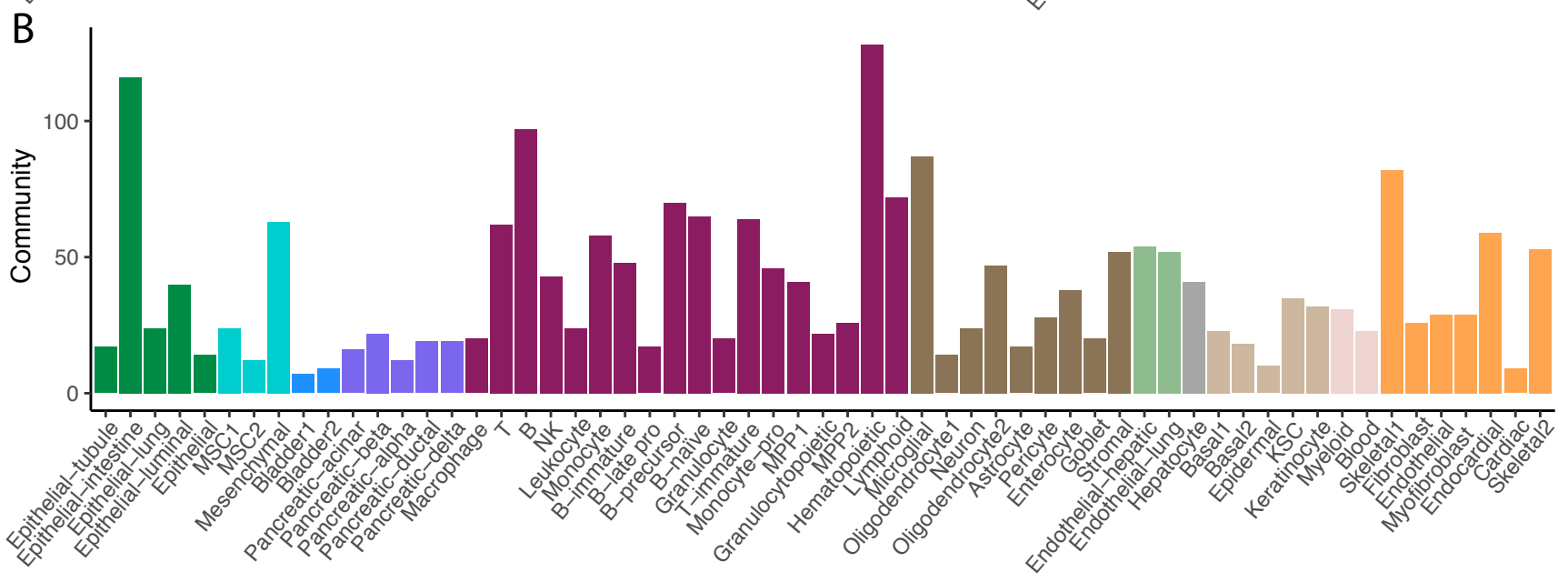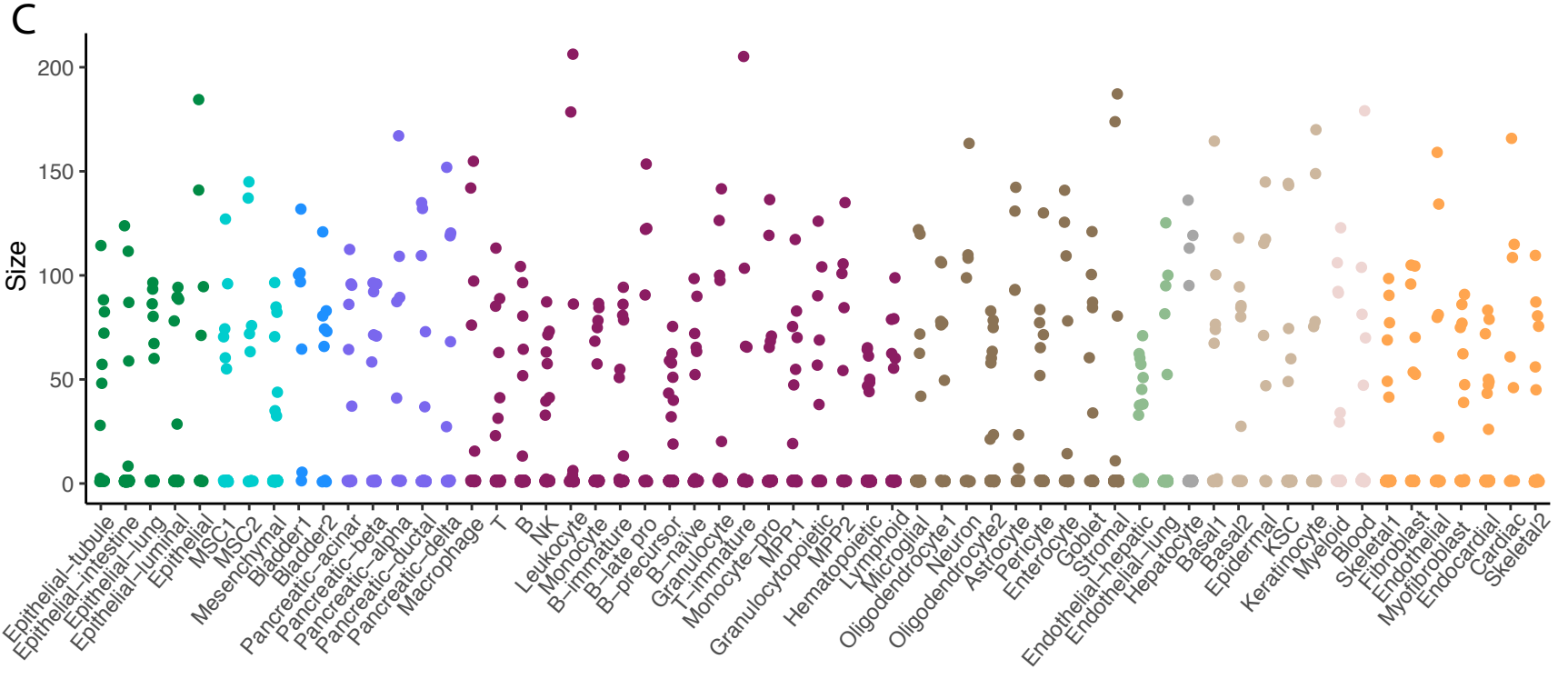

Supplement: Supplementary Figure S4 — Network statistics of the gene co-expression networks identified by scLink on the Tabula Muris data A. Degree of genes in the cell-type-specific gene networks. B. Number of communities (identified by Louvain algorithm) in the cell-type-specific gene networks. C. Sizes of communities (identified by Louvain algorithm) in the cell-type-specific gene networks. [file mmc5.pdf]

A

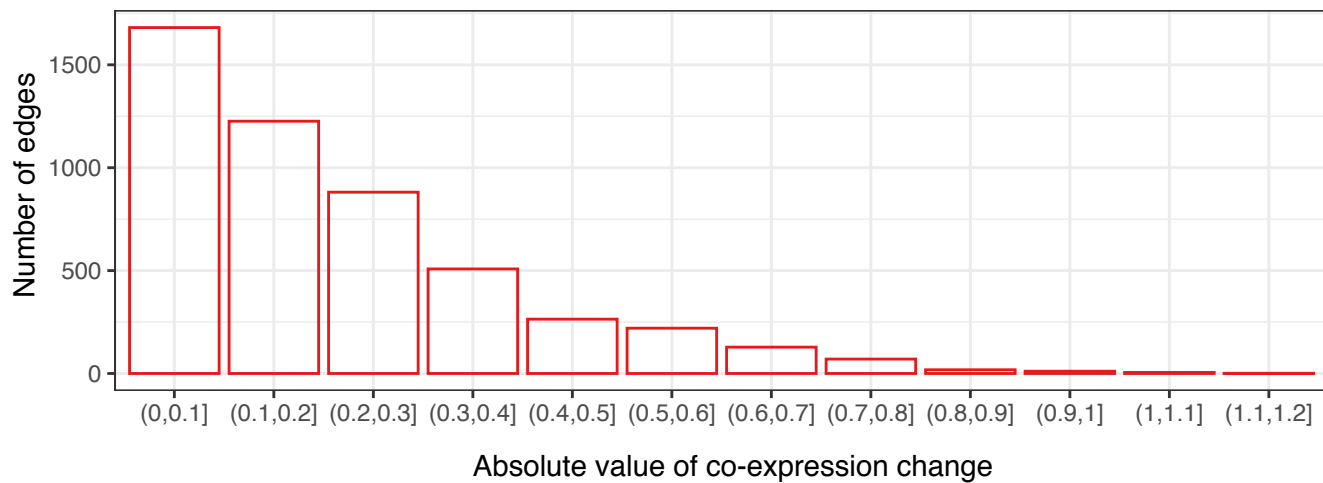

B

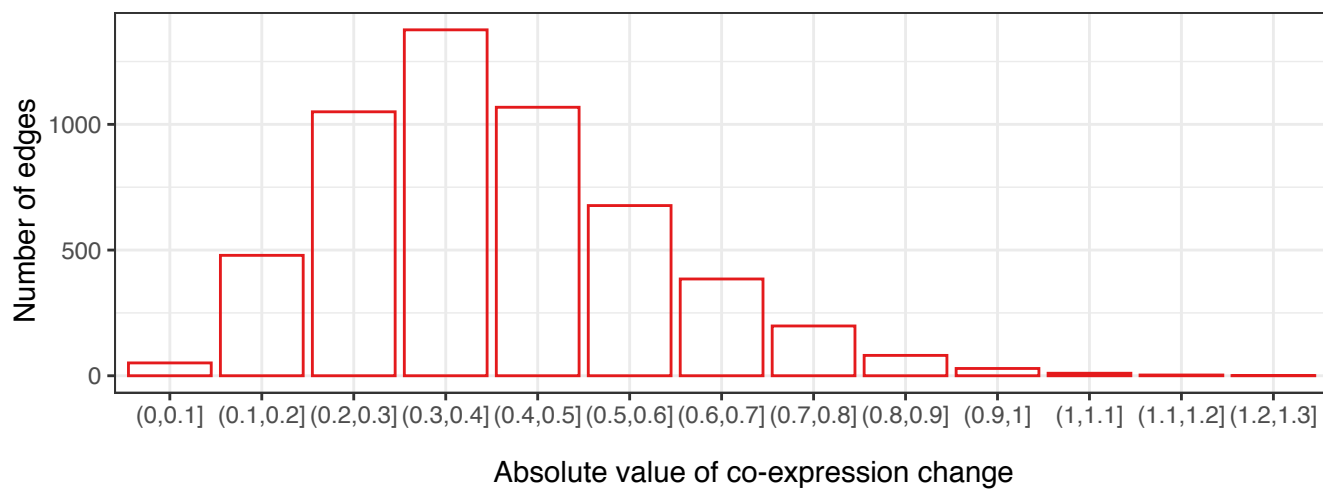

C

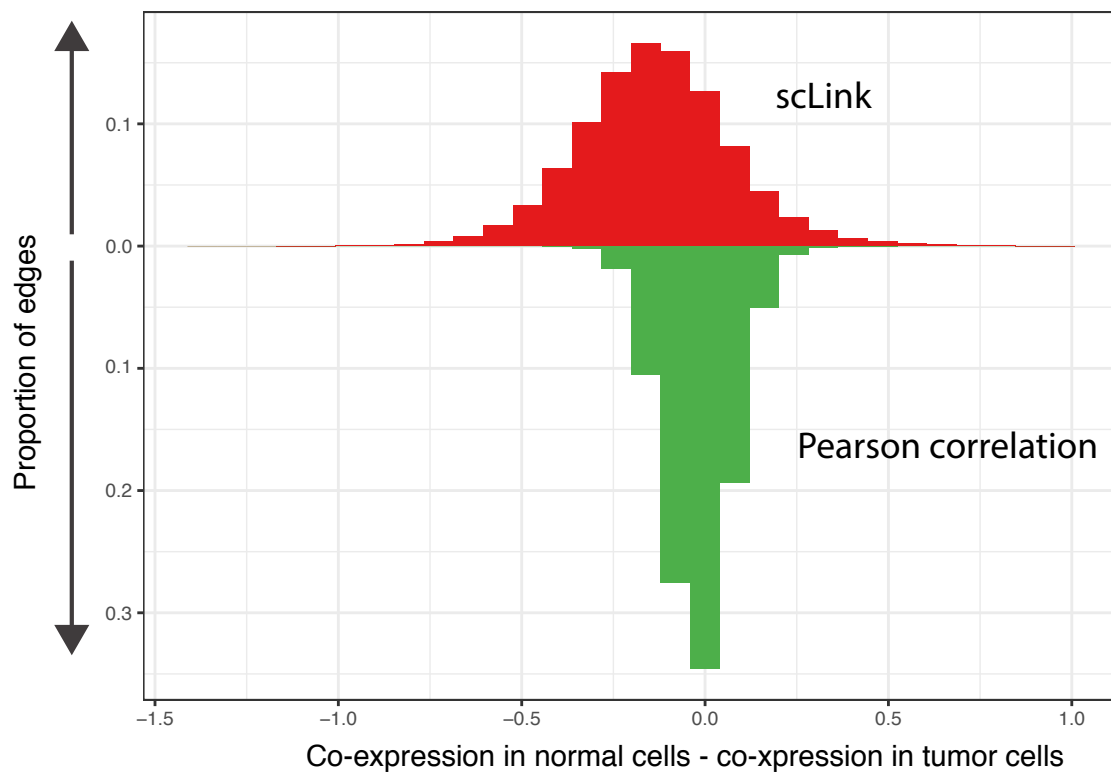

Supplement: Supplementary Figure S5 — Summary of differential edges identified in the breast cancer application A. Number of differential edges only present in normal immune cells, categorized by the absolute value of scLink’s co-expression change between normal and tumor conditions. B. Number of edges only present in tumor cells, categorized by the absolute value of scLink's co-expression change between normal and tumor conditions. C. Distribution of co-expression changes between normal and tumor cells as measured by scLink's correlation or the Pearson correlation coefficients. [file mmc6.pdf]

A

### Significance of 453 edges in normal cells

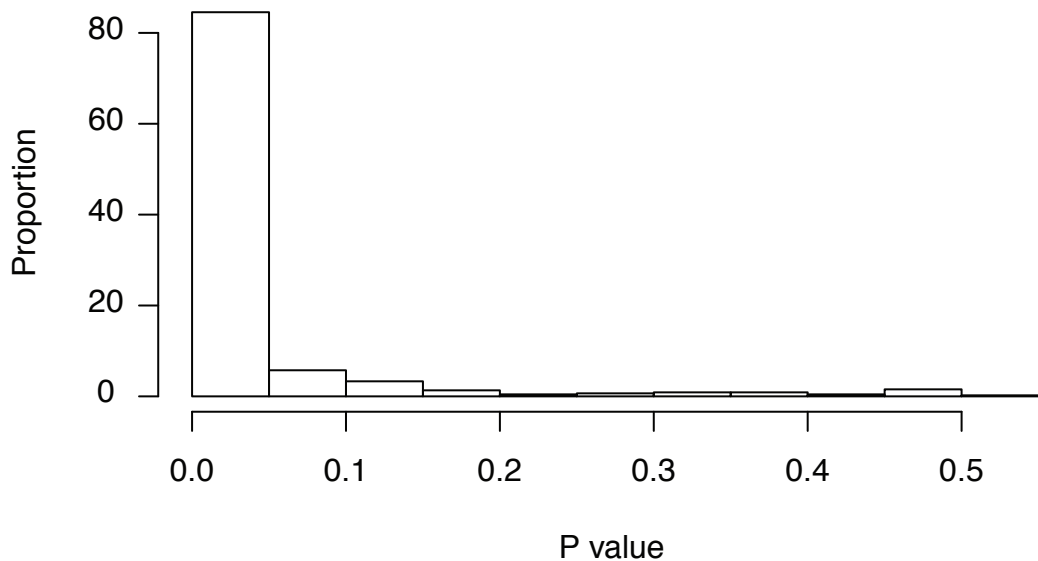

B

### Significance of 1384 edges in tumor cells

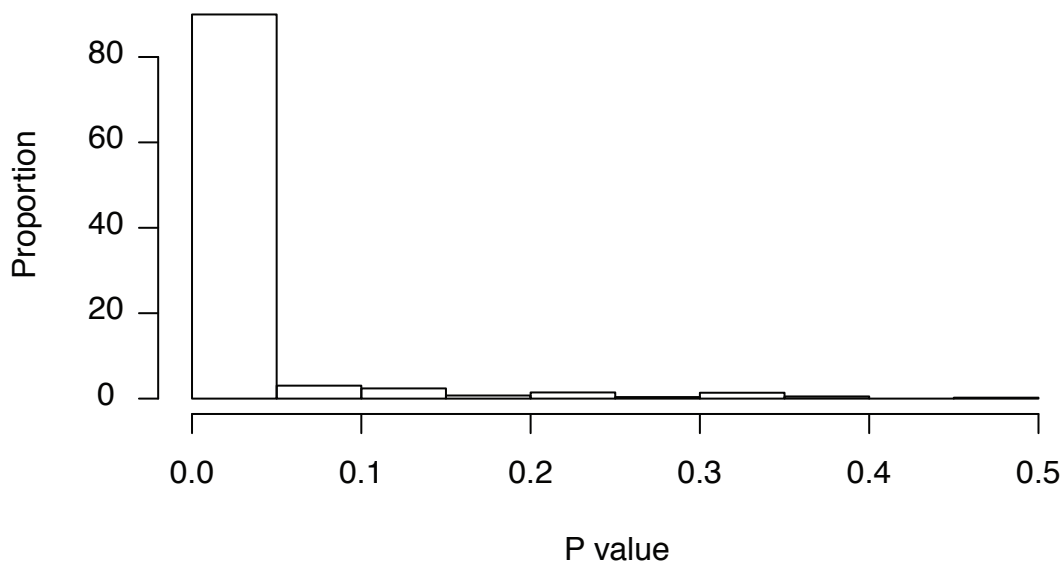

Supplement: Supplementary Figure S6 — Assessing the statistical significance of scLink correlation A. Bootstrap P values of the scLink correlation for the 453 differential edges only present in the normal sample. B. Bootstrap P values of the scLink correlation for the 1384 differential edges only present in the tumor sample. [file mmc7.pdf]

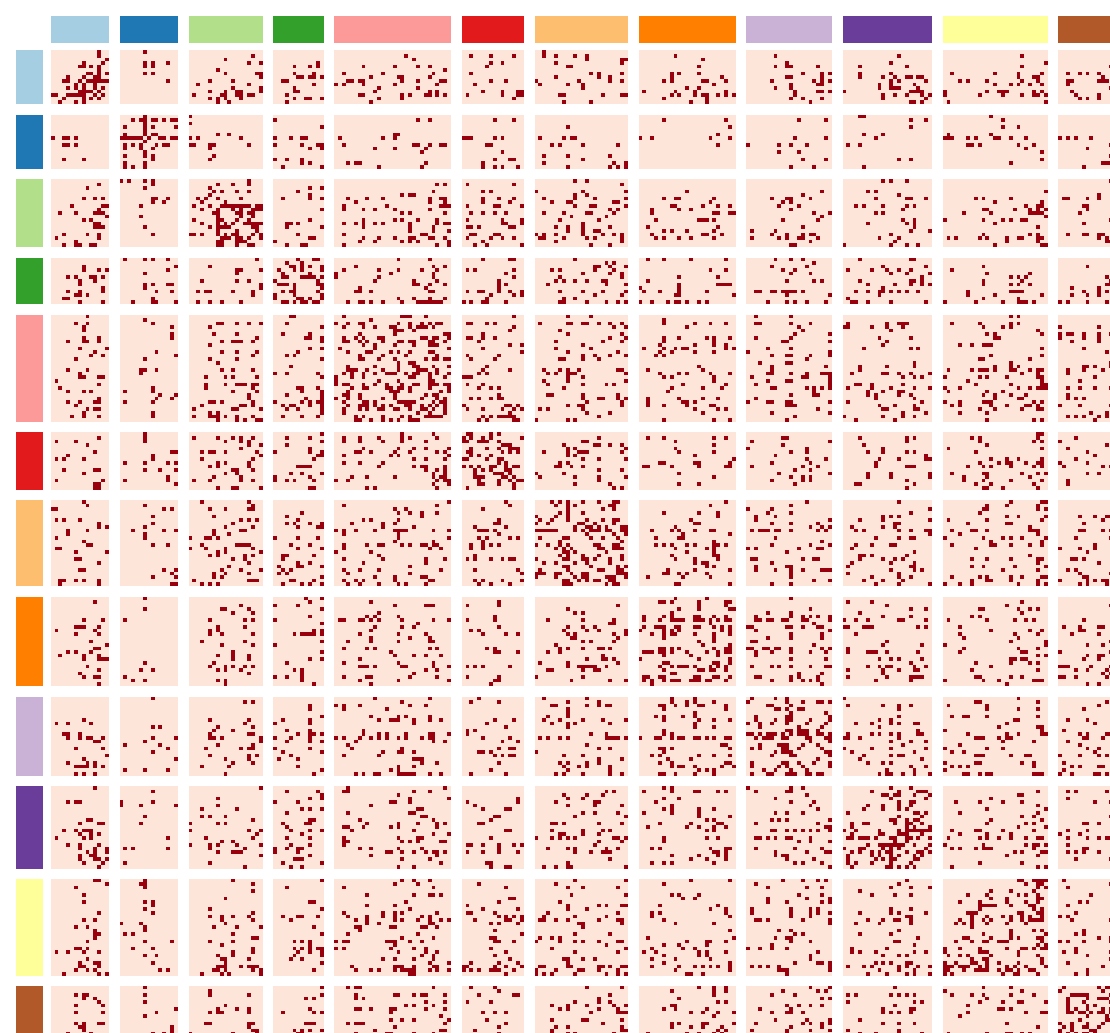

Edge

No edge

Module

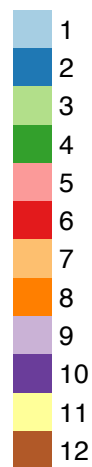

Supplement: Supplementary Figure S7 — scLink identifies gene modules in immune cells of normal sample Hierarchical clustering of genes was performed using the (1-|partial correlation matrix|) as the distance matrix. Modules were assigned by cutting the hierarchical dendrogram at the 90% percentile of all partial correlation coefficients. Only modules with more than 10 genes are displayed. The heatmap value denotes whether scLink identifies an edge for each pair of genes. [file mmc8.pdf]

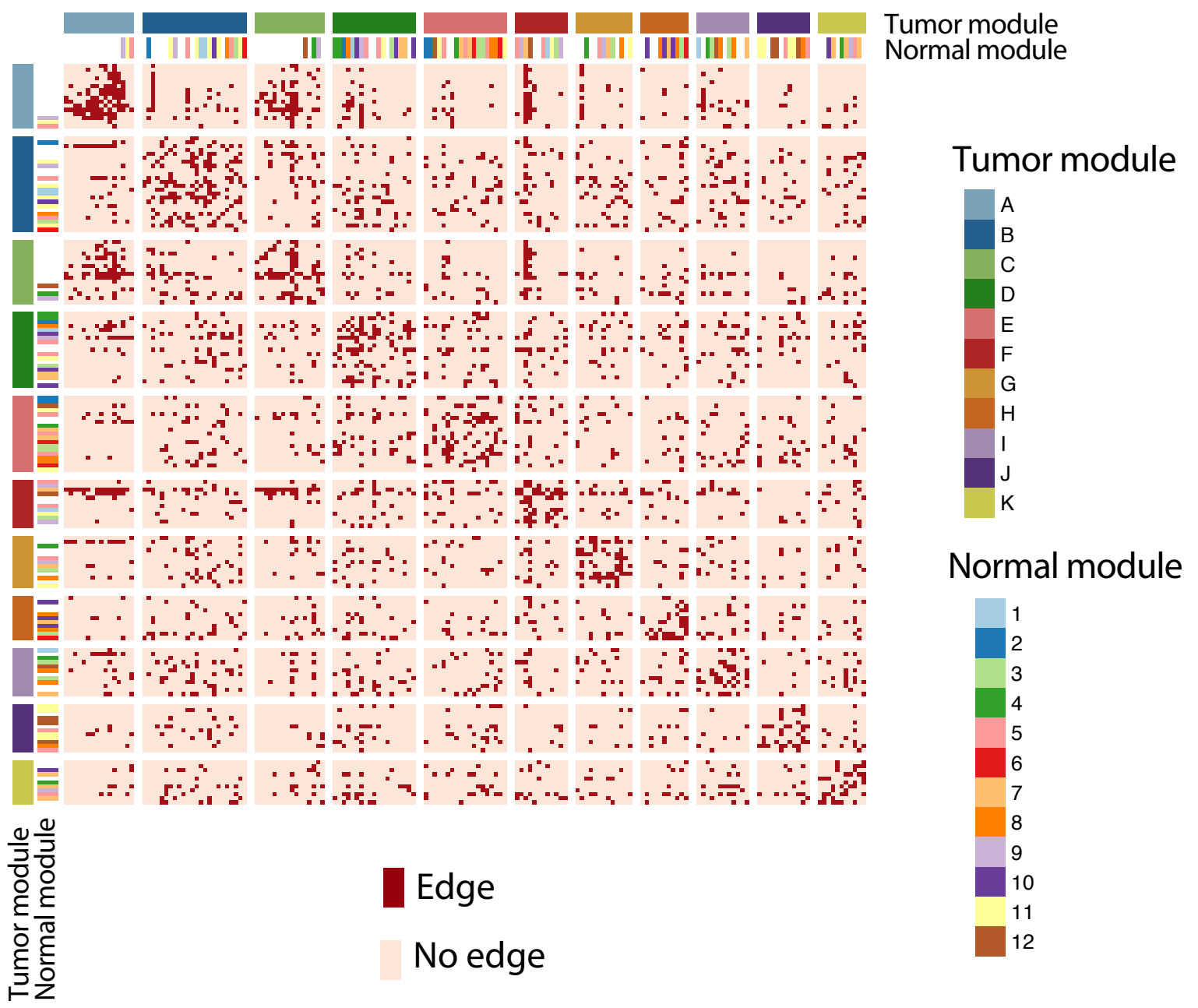

Supplement: Supplementary Figure S8 — scLink identifies gene modules in immune cells of tumor sample Hierarchical clustering of genes was performed using the (1-|partial correlation matrix|) as the distance matrix. Modules were assigned by cutting the hierarchical dendrogram at the 90% percentile of all partial correlation coefficients. Only modules with more than 10 genes are displayed. The heatmap value denotes whether scLink identifies an edge for each pair of genes in the tumor cells. [file mmc9.pdf]

## Normal

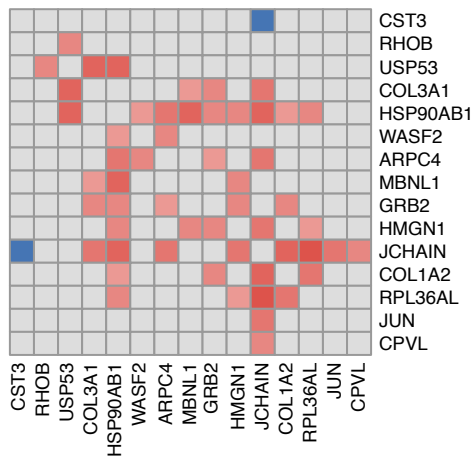

## Corrected correlation by scLink

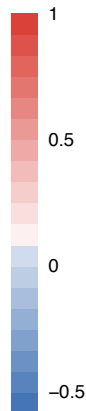

## Tumor

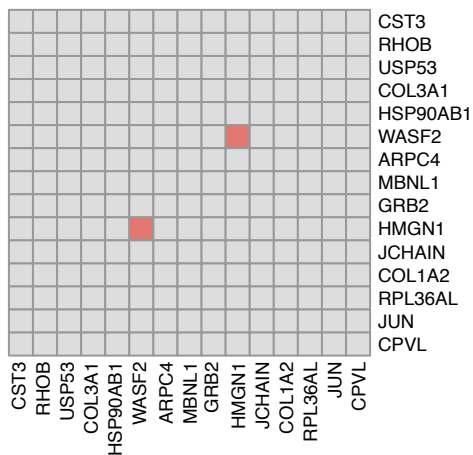

Edge

No edge

Supplement: Supplementary Figure S9 — Gene network is rewired in tumor cells The correlation matrices of a 15-gene module identified by scLink from the normal sample are displayed in the heatmaps. Only the correlation measures for identified edges in the normal or tumor sample are shown. Otherwise, the corresponding entries are colored in gray. [file mmc10.pdf]

A

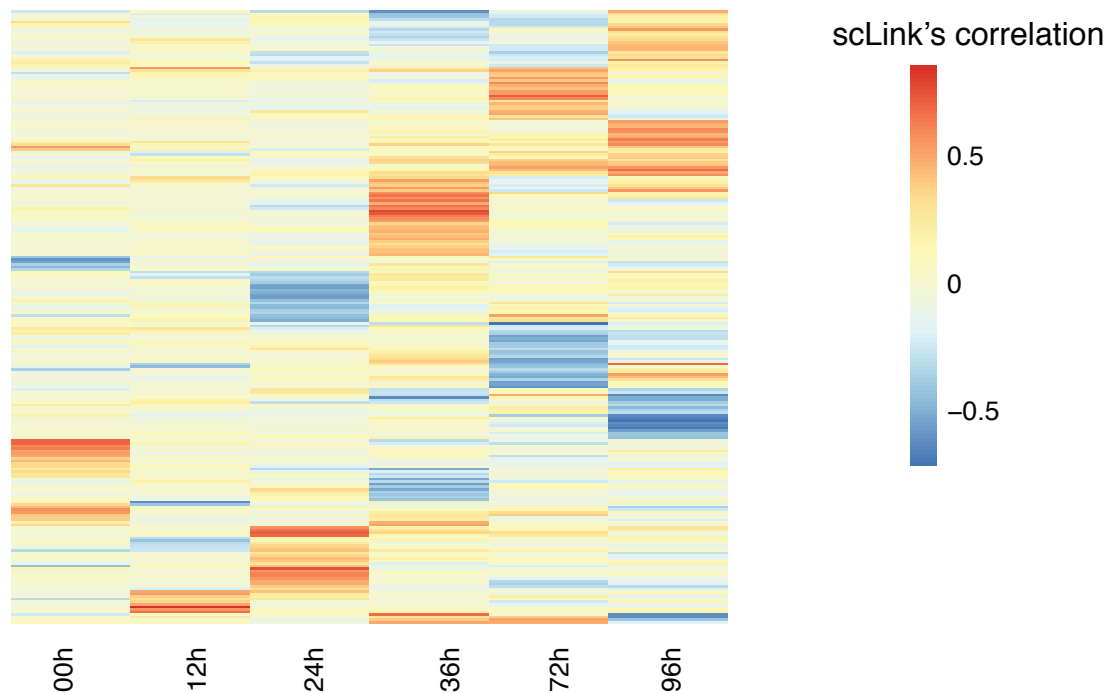

B

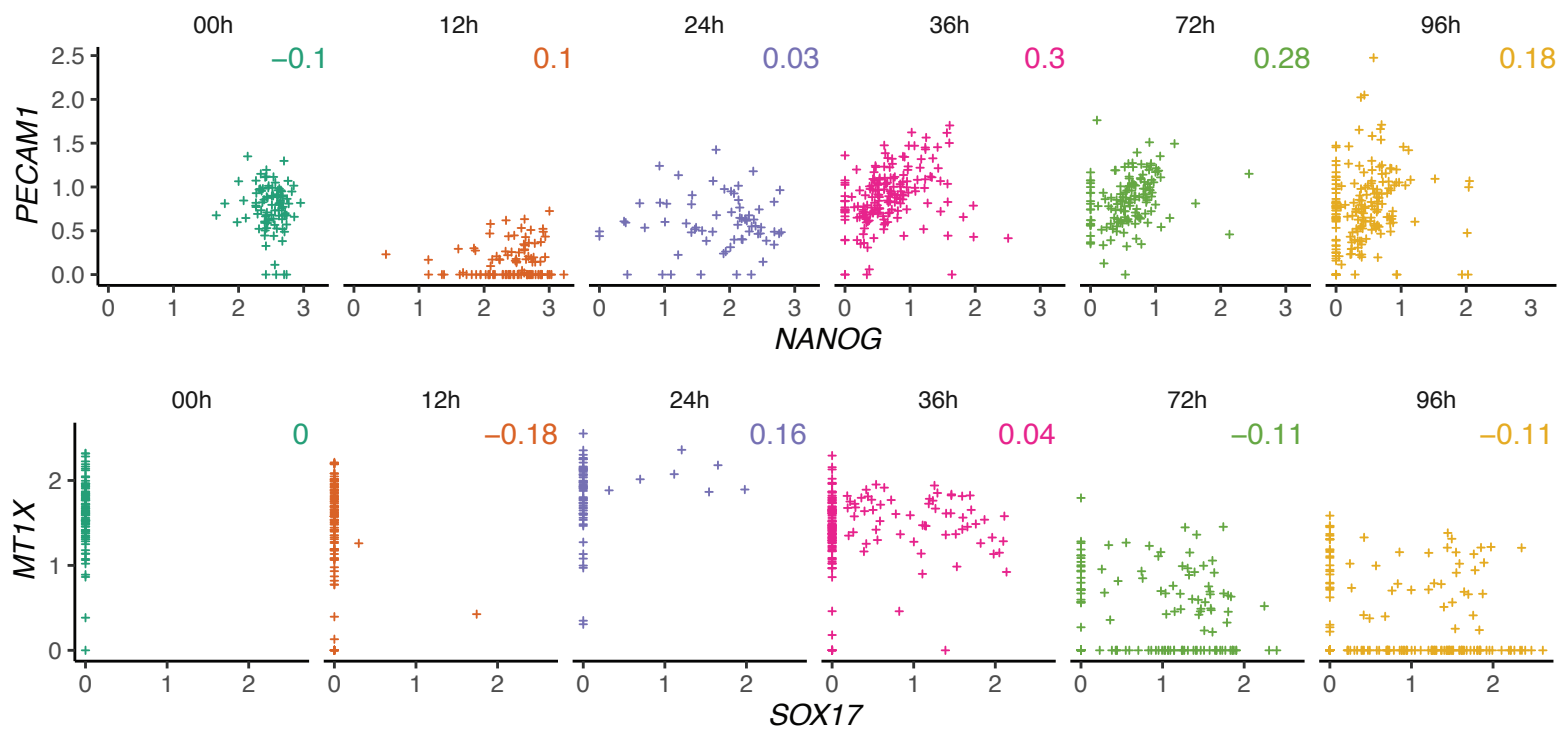

Supplement: Supplementary Figure S10 — Application of scLink on time-course scRNA-seq data A. The correlation calculated by scLink for 240 gene pairs at different time points. These 240 gene pairs have a correlation change greater than 0.5 along the time course of definitive endoderm differentiation. B. Log10-transformed gene expression of PECAM1 and NANOG, MT1X and SOX17 at different time points in the differentiation process. Displayed numbers are the Pearson correlation coefficients. [file mmc11.pdf]

scLink's correlation

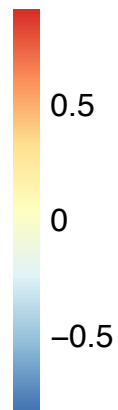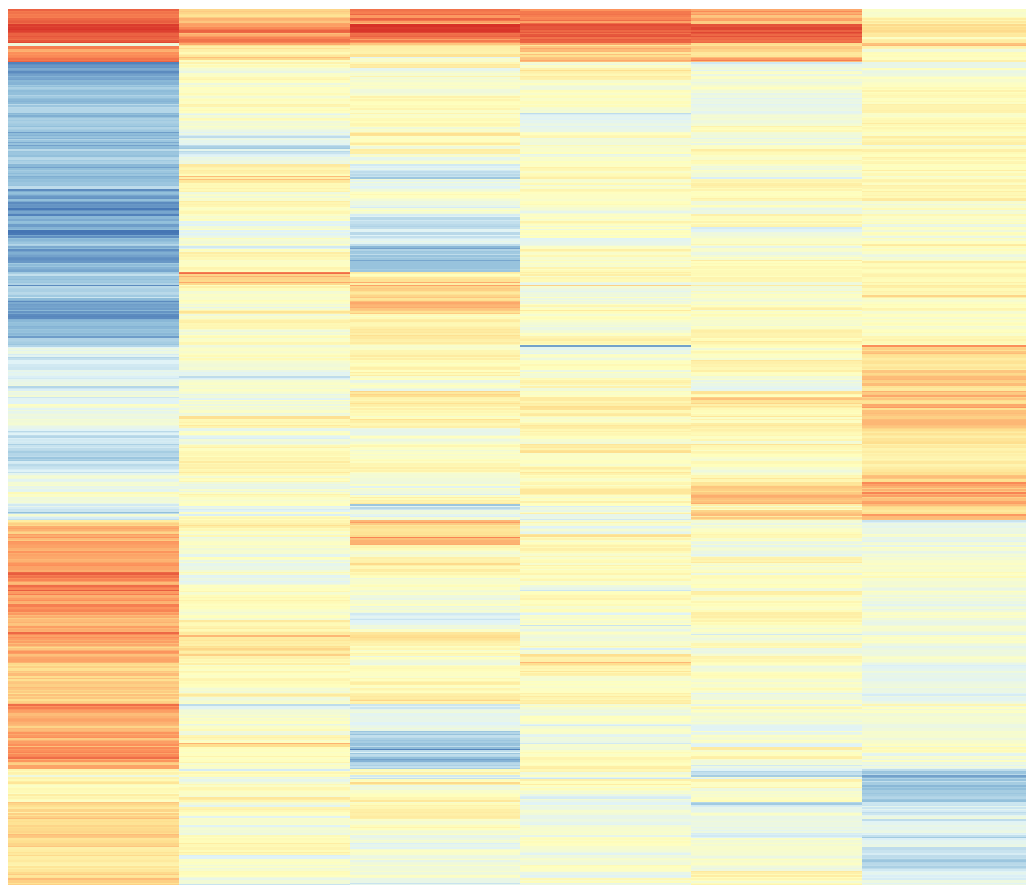

00h

12h

24h

36h

72h

96h

Supplement: Supplementary Figure S11 — The correlation by scLink of 595 differential edges between 0 h and 96 h These 595 gene pairs only have edges in either the 0 h network or the 96 h network, and have a correlation change greater than 0.5 along the time course of endoderm differentiation. [file mmc12.pdf]

A

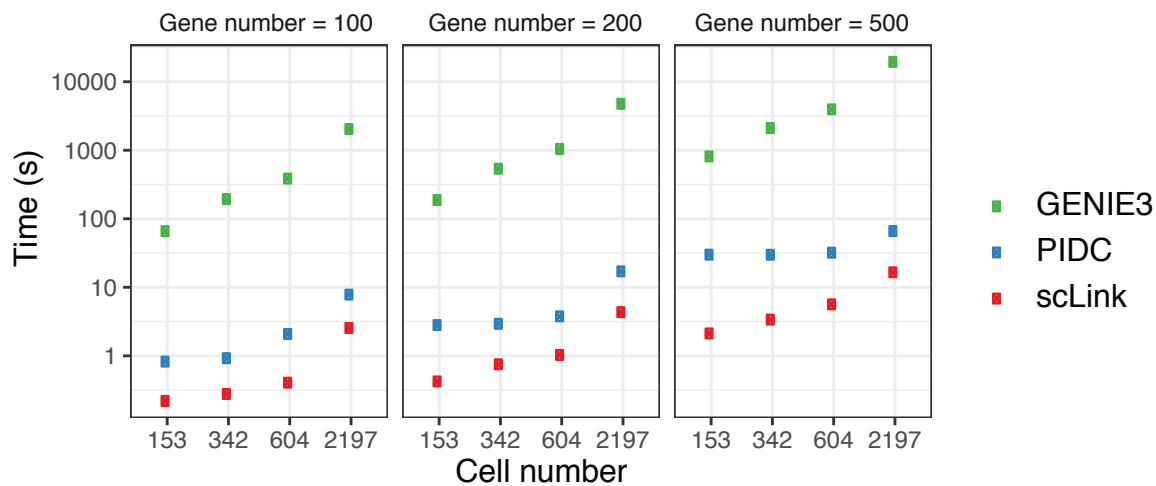

B

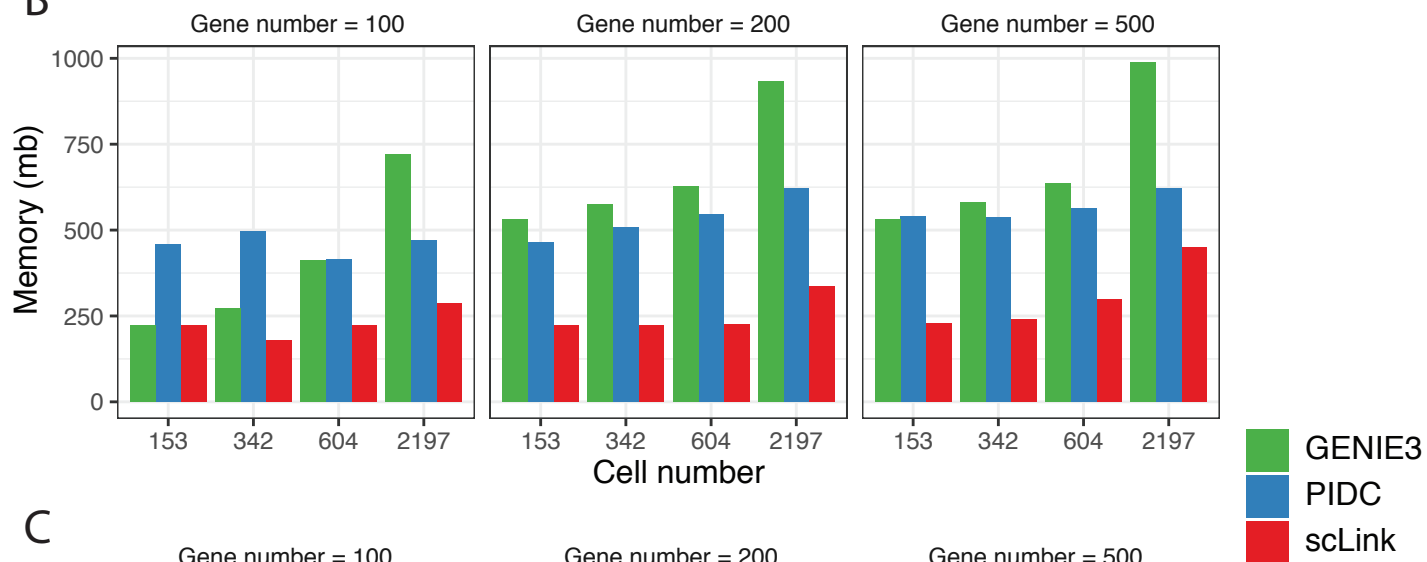

C

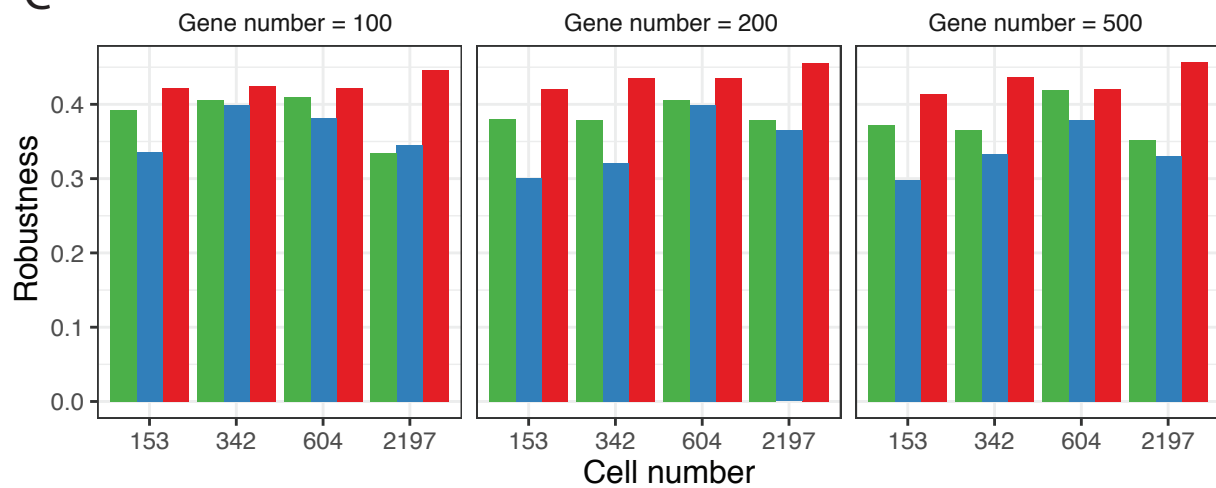

Supplement: Supplementary Figure S14 — Evaluation of computational performance based on Smart-Seq2 data A. Computation time summarized in seconds. B. Memory usage summarized in megabytes. C. Robustness score. [file mmc15.pdf]

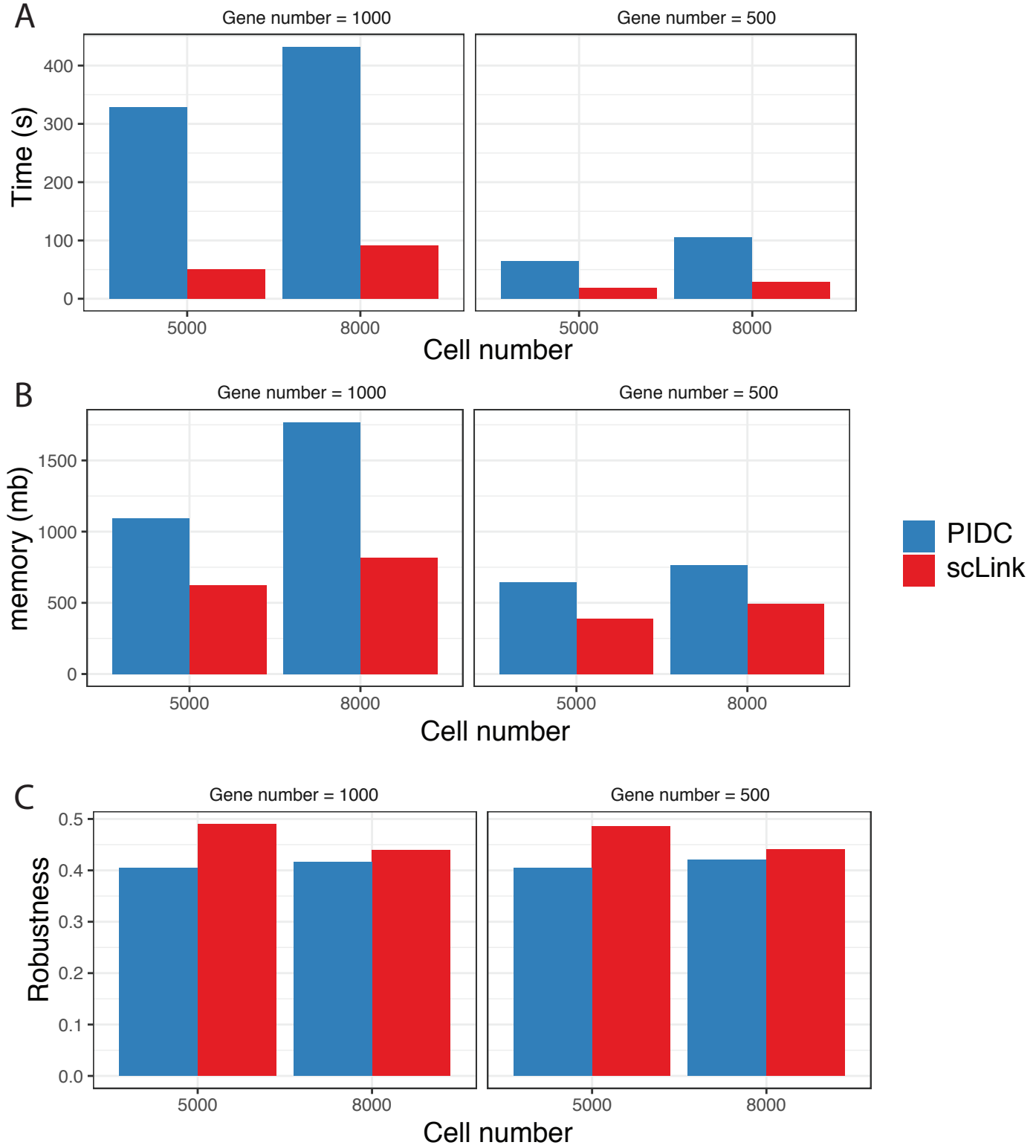

Supplement: Supplementary Figure S15 — Evaluation of computational performance based on 10x Genomics data A. Computation time summarized in seconds. B. Memory usage summarized in megabytes. C. Robustness score. [file mmc16.pdf]
