# Supplementary material for: scLink: Inferring Sparse Gene Co-expression Networks from Single-cell Expression Data
Source: Genomics Proteomics Bioinformatics. 2021 Jul 10;19(3):475–92. doi: 10.1016/j.gpb.2020.11.006 (PMC8896229; doi:10.1016/j.gpb.2020.11.006)
Supplement: Supplementary Figure S12 — GO enrichment analysis of scLink’s results on time-course scRNA-seq data A. Top enriched GO terms in genes with higher degrees at 0 h than at 96 h. We used all the genes whose degrees at 0 h are at least 20 greater than their degrees at 96 h. B. Top enriched GO terms in genes with higher degrees at 96 h than at 0 h. We used all the genes whose degrees at 96 h are at least 20 greater than their degrees at 0 h. [file mmc13.pdf]

A

GO ID	*P* value	Description
GO:0051304	***	chromosome separation
GO:0007091	***	metaphase/anaphase transition of mitotic cell cycle
GO:0010965	***	regulation of mitotic sister chromatid separation
GO:0030071	***	regulation of mitotic metaphase/anaphase transition
GO:0044784	***	metaphase/anaphase transition of cell cycle
GO:0051306	***	mitotic sister chromatid separation
GO:1902099	***	regulation of metaphase/anaphase transition of cell cycle
GO:1905818	***	regulation of chromosome separation
GO:0033047	***	regulation of mitotic sister chromatid segregation
GO:0007093	***	mitotic cell cycle checkpoint
GO:0000070	***	mitotic sister chromatid segregation
GO:0044772	***	mitotic cell cycle phase transition
GO:0000075	***	cell cycle checkpoint
GO:0000819	***	sister chromatid segregation
GO:0033045	***	regulation of sister chromatid segregation
GO:0044770	***	cell cycle phase transition

B

GO ID	*P* value	Description
GO:0048708	***	astrocyte differentiation
GO:0034405	**	response to fluid shear stress
GO:0050793	**	regulation of developmental process
GO:0051239	**	regulation of multicellular organismal process
GO:2000026	**	regulation of multicellular organismal development
GO:0045595	**	regulation of cell differentiation
GO:0003008	**	system process
GO:0006814	*	sodium ion transport
GO:0010001	*	glial cell differentiation
GO:0045667	*	regulation of osteoblast differentiation

* *P* value < 0.01, ** *P* value < 0.001, *P* value < 0.0001
