# Supplementary material for: scLink: Inferring Sparse Gene Co-expression Networks from Single-cell Expression Data
Source: Genomics Proteomics Bioinformatics. 2021 Jul 10;19(3):475–92. doi: 10.1016/j.gpb.2020.11.006 (PMC8896229; doi:10.1016/j.gpb.2020.11.006)
Supplement: Supplementary Figure S13 — GO enrichment analysis of Pearson correlation networks for time-course scRNA-seq data A. Comparison of genes’ degree changes from 0 h to 96 h in scLink and Pearson correlation networks. B. Top enriched GO terms in genes with higher degrees at 0 h than at 96 h. We used all the genes whose degrees at 0 h are at least 20 greater than their degrees at 96 h. C. Top enriched GO terms in genes with higher degrees at 96 h than at 0 h. We used all the genes whose degrees at 96 h are at least 20 greater than their degrees at 0 h. [file mmc14.pdf]

A

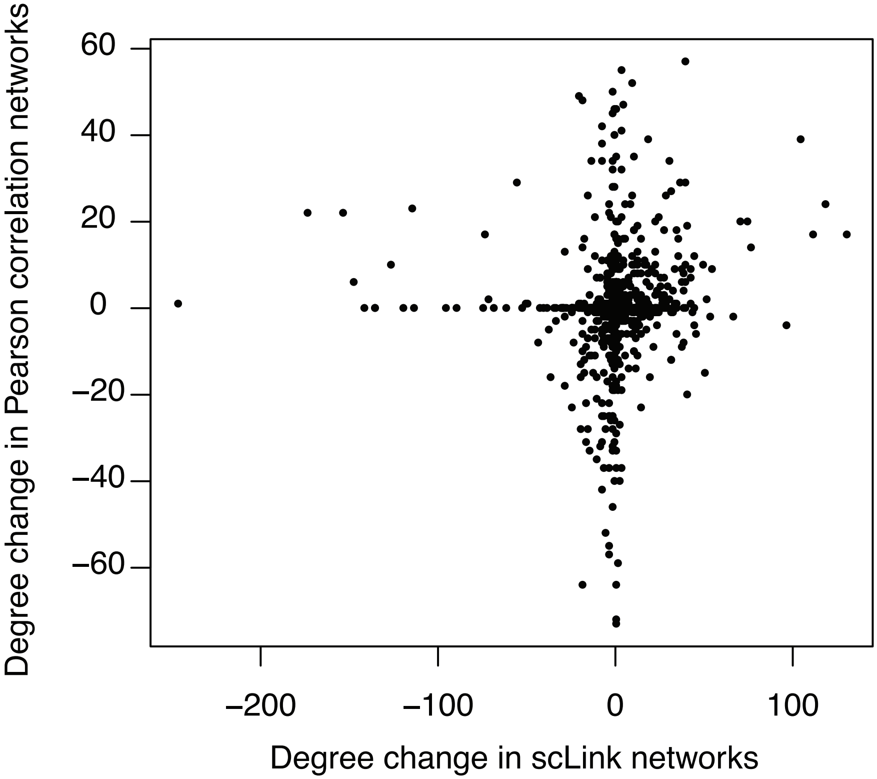

B

GO ID	*P* value	Description
GO:0006614	***	SRP-dependent cotranslational protein targeting to membrane
GO:0000184	***	nuclear-transcribed mRNA catabolic process, nonsense-mediated decay
GO:0006613	***	cotranslational protein targeting to membrane
GO:0045047	***	protein targeting to ER
GO:0072599	***	establishment of protein localization to endoplasmic reticulum
GO:0006612	***	protein targeting to membrane
GO:0070972	***	protein localization to endoplasmic reticulum
GO:0000956	***	nuclear-transcribed mRNA catabolic process
GO:0006413	***	translational initiation
GO:0090150	***	establishment of protein localization to membrane

C

GO ID	*P* value	Description
GO:1900118	***	negative regulation of execution phase of apoptosis
GO:1900117	**	regulation of execution phase of apoptosis
GO:1900115	**	extracellular regulation of signal transduction
GO:1900116	**	extracellular negative regulation of signal transduction
GO:0097194	*	execution phase of apoptosis

* *P* value < 0.01, ** *P* value < 0.001, *P* value < 0.0001
