# Supplementary material for: scLink: Inferring Sparse Gene Co-expression Networks from Single-cell Expression Data
Source: Genomics Proteomics Bioinformatics. 2021 Jul 10;19(3):475–92. doi: 10.1016/j.gpb.2020.11.006 (PMC8896229; doi:10.1016/j.gpb.2020.11.006)

scLink About Example Figures Downloads

Here is an example showing the results available at this app. You can select your cell type and gene names of interest on the Figures page.

Example cell type:

T cell

Example gene names:

Hsp90b1 Canx Calr Tap2 H2-T23 Rhoa Tapbp H2-Q6 Pdia3

Heatmap Network Graph Matrix

Heatmap of scLink's correlation matrix

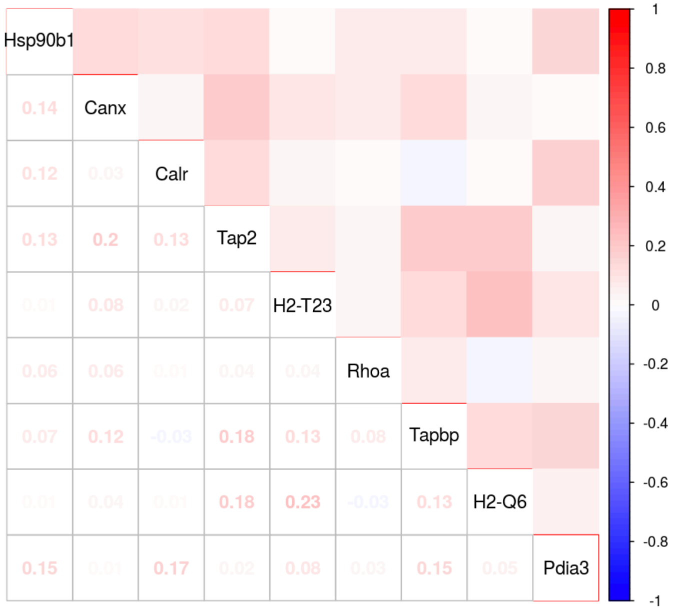

Supplement: Supplementary Figure S16 — The scLink web page provides an interactive application to demonstrate the usage and results of scLink [file mmc17.pdf]
