# Supplementary material for: scLink: Inferring Sparse Gene Co-expression Networks from Single-cell Expression Data
Source: Genomics Proteomics Bioinformatics. 2021 Jul 10;19(3):475–92. doi: 10.1016/j.gpb.2020.11.006 (PMC8896229; doi:10.1016/j.gpb.2020.11.006)
Supplement: Supplementary Table S1 — Proportions of the computationally identified TF-gene edges that were previously discovered in ChIP-seq experiments [file mmc18.docx]

**Table S1 Proportions of the computationally identified TF-gene edges that were previously discovered in ChIP-seq experiments**

| **Method** | **Min** | $\boldsymbol{Q}_{\boldsymbol{1}}$ | **Median** | **Mean** | $\boldsymbol{Q}_{\boldsymbol{3}}$ | **Max** |
| --- | --- | --- | --- | --- | --- | --- |
| scLink | 15.6% (6) | **48.8%** (105) | **59.3%** (153) | **56.4%** (174) | **69.8%** (245) | **89.5%** (502) |
| glasso-f | 10.0% (2) | 47.3% (79) | 56.4% (147) | 55.2% (160) | 68.6% (231) | **96.7%** (393) |
| glasso-r | 7.7% (4) | 46.5% (65) | 58.1% (124) | 52.3% (149) | 66.5% (228) | 87.1% (377) |
| Spearman | **20.0%** (23) | 46.3% (114) | 53.6% (164) | 53.0% (163) | 61.4% (202) | 81.0% (422) |
| Pearson | **19.7%** (24) | 47.5% (121) | 53.0% (196) | 53.1% (194) | 61.3% (246) | 80.5% (470) |
| PIDC | 17.8% (8) | **50.1%** (152) | **63.5%** (191) | **60.3%** (210) | **71.9%** (267) | 86.8% (509) |

*Note*: Proportions were summarized using minimum, 1st quartile, median, mean, 3rd quartile, and maximum values across 59 cell types in the Tabula Muris data. The total numbers of computationally identified TF-gene edges, summarized using minimum (Min), 1st quartile ($Q_{1}$), median, mean, 3rd quartile ($Q_{3}$), and maximum (Max) values across 59 cell types in the *Tabula Muris* data were shown in the parentheses.
