# Supplementary material for: scLink: Inferring Sparse Gene Co-expression Networks from Single-cell Expression Data
Source: Genomics Proteomics Bioinformatics. 2021 Jul 10;19(3):475–92. doi: 10.1016/j.gpb.2020.11.006 (PMC8896229; doi:10.1016/j.gpb.2020.11.006)
Supplement: Supplementary Table S2 — Network analysis of the Tabula Muris data using correlation-based, PIDC, and glasso-based methods [file mmc19.docx]

**Table S2 Network analysis of the Tabula Muris data using correlation-based, PIDC, and glasso-based methods**

|  |  | **T cells** | **Muscle stem cells** | **Beta cells** |
| --- | --- | --- | --- | --- |
| **Pearson correlation** | Number of genes | 105 | 117 | 71 |
|  | Number of ribosomal protein genes | 75 | 74 | 18 |
|  | Proportion of edges shared with scLink | 2.9% | 0.1% | 43.0% |
| **Spearman’s correlation** | Number of genes | 99 | 96 | 90 |
|  | Number of ribosomal protein genes | 75 | 74 | 19 |
|  | Proportion of edges shared with scLink | 2.7% | 0.2% | 46.5% |
| **PIDC** | Number of genes | 91 | 116 | 37 |
|  | Number of ribosomal protein genes | 74 | 74 | 2 |
|  | Proportion of edges shared with scLink | 3.2% | 0.2% | 38.8% |
| **glasso-f** | Number of genes | 46 | 17 | 45 |
|  | Number of ribosomal protein genes | 2 | 0 | 15 |
|  | Proportion of edges shared with scLink | 33.3% | 70.4% | 51.0% |
| **glasso-r** | Number of genes | 23 | 18 | 30 |
|  | Number of ribosomal protein genes | 0 | 0 | 17 |
|  | Proportion of edges shared with scLink | 91.2% | 100% | 93.8% |

*Note*: For each of the inferred networks, we investigated its largest connected component and summarized the gene number and overlap with scLink’s edges.
