# Supplementary material for: scLink: Inferring Sparse Gene Co-expression Networks from Single-cell Expression Data
Source: Genomics Proteomics Bioinformatics. 2021 Jul 10;19(3):475–92. doi: 10.1016/j.gpb.2020.11.006 (PMC8896229; doi:10.1016/j.gpb.2020.11.006)
Supplement: Supplementary Table S3 — GO terms enriched in the largest connected components of Pearson correlation-based networks constructed from Tabula Muris data [file mmc20.docx]

**Table S3 GO terms enriched in the largest connected components of Pearson correlation-based networks constructed from Tabula Muris data**

| **GO term enriched in the largest connected component in T cells** | | |
| --- | --- | --- |
| ID | Description | Adjusted *P* |
| GO:0006412 | translation | 2.39E-43 |
| GO:0043043 | peptide biosynthetic process | 2.39E-43 |
| GO:0043604 | amide biosynthetic process | 7.39E-43 |
| GO:0006518 | peptide metabolic process | 2.48E-40 |
| GO:0043603 | cellular amide metabolic process | 2.61E-39 |
| GO:1901566 | organonitrogen compound biosynthetic process | 1.21E-30 |
| GO:0042254 | ribosome biogenesis | 8.20E-20 |
| GO:0044267 | cellular protein metabolic process | 5.28E-18 |
| GO:0034645 | cellular macromolecule biosynthetic process | 1.51E-16 |
| GO:0022613 | ribonucleoprotein complex biogenesis | 1.51E-16 |
| **GO terms enriched in the largest connected component in muscle cells** | | |
| ID | Description | Adjusted *P* |
| GO:0006412 | translation | 5.67E-43 |
| GO:0043043 | peptide biosynthetic process | 1.30E-42 |
| GO:0043604 | amide biosynthetic process | 3.85E-42 |
| GO:0006518 | peptide metabolic process | 4.20E-39 |
| GO:0043603 | cellular amide metabolic process | 1.17E-38 |
| GO:1901566 | organonitrogen compound biosynthetic process | 8.78E-35 |
| GO:0042254 | ribosome biogenesis | 9.98E-18 |
| GO:0044267 | cellular protein metabolic process | 9.10E-17 |
| GO:0022613 | ribonucleoprotein complex biogenesis | 1.75E-15 |
| GO:0019538 | protein metabolic process | 6.56E-15 |
| **GO terms enriched in the largest connected component in beta cells** | | |
| ID | Description | Adjusted *P* |
| GO:0006518 | peptide metabolic process | 1.06E-07 |
| GO:0006412 | translation | 6.03E-07 |
| GO:0043603 | cellular amide metabolic process | 6.03E-07 |
| GO:0043043 | peptide biosynthetic process | 6.63E-07 |
| GO:0043604 | amide biosynthetic process | 3.08E-06 |
| GO:0042255 | ribosome assembly | 0.0020 |
| GO:1901566 | organonitrogen compound biosynthetic process | 0.0036 |
| GO:0002181 | cytoplasmic translation | 0.0055 |
| GO:0019538 | protein metabolic process | 0.0056 |
| GO:0070972 | protein localization to endoplasmic reticulum | 0.0063 |

*Note*: A significance level of 0.01 was applied to the FDR-adjusted *P* values. Only the most significant 10 GO terms were shown if more than 10 were enriched.
