# Supplementary material for: scLink: Inferring Sparse Gene Co-expression Networks from Single-cell Expression Data
Source: Genomics Proteomics Bioinformatics. 2021 Jul 10;19(3):475–92. doi: 10.1016/j.gpb.2020.11.006 (PMC8896229; doi:10.1016/j.gpb.2020.11.006)
Supplement: Supplementary Table S4 — GO terms enriched in the largest connected components of Spearman’s correlation-based networks constructed from Tabula Muris data [file mmc21.docx]

**Table S4 GO terms enriched in the largest connected components of Spearman’s correlation-based networks constructed from Tabula Muris data**

| **GO terms enriched in the largest connected component in T cells** | | |
| --- | --- | --- |
| ID | Description | Adjusted *P* |
| GO:0006412 | translation | 1.12E-41 |
| GO:0043043 | peptide biosynthetic process | 1.12E-41 |
| GO:0043604 | amide biosynthetic process | 3.03E-41 |
| GO:0006518 | peptide metabolic process | 4.65E-39 |
| GO:0043603 | cellular amide metabolic process | 4.03E-38 |
| GO:1901566 | organonitrogen compound biosynthetic process | 9.64E-29 |
| GO:0042254 | ribosome biogenesis | 5.97E-21 |
| GO:0034645 | cellular macromolecule biosynthetic process | 7.75E-19 |
| GO:0044267 | cellular protein metabolic process | 9.22E-19 |
| GO:0022613 | ribonucleoprotein complex biogenesis | 8.71E-18 |
| **GO terms enriched in the largest connected component in muscle cells** | | |
| ID | Description | Adjusted *P* |
| GO:0006412 | translation | 2.70E-54 |
| GO:0043043 | peptide biosynthetic process | 6.52E-54 |
| GO:0043604 | amide biosynthetic process | 2.02E-53 |
| GO:0006518 | peptide metabolic process | 1.01E-48 |
| GO:0043603 | cellular amide metabolic process | 2.85E-48 |
| GO:1901566 | organonitrogen compound biosynthetic process | 1.34E-41 |
| GO:0042254 | ribosome biogenesis | 4.69E-21 |
| GO:0044267 | cellular protein metabolic process | 2.15E-18 |
| GO:0034645 | cellular macromolecule biosynthetic process | 2.39E-17 |
| GO:0009059 | macromolecule biosynthetic process | 9.97E-17 |
| **GO terms enriched in the largest connected component in beta cells** | | |
| ID | Description | Adjusted *P* |
| GO:0006518 | peptide metabolic process | 9.01E-07 |
| GO:0043603 | cellular amide metabolic process | 9.93E-06 |
| GO:0006412 | translation | 0.0001 |
| GO:0043043 | peptide biosynthetic process | 0.0002 |
| GO:0019538 | protein metabolic process | 0.0002 |
| GO:0043604 | amide biosynthetic process | 0.0006 |

*Note*: A significance level of 0.01 was applied to the FDR-adjusted *P* values. Only the most significant 10 GO terms were shown if more than 10 were enriched.
