# Supplementary material for: scLink: Inferring Sparse Gene Co-expression Networks from Single-cell Expression Data
Source: Genomics Proteomics Bioinformatics. 2021 Jul 10;19(3):475–92. doi: 10.1016/j.gpb.2020.11.006 (PMC8896229; doi:10.1016/j.gpb.2020.11.006)
Supplement: Supplementary Table S5 — GO terms enriched in the largest connected components of PIDC networks constructed from Tabula Muris data [file mmc22.docx]

**Table S5 GO terms enriched in the largest connected components of PIDC networks constructed from Tabula Muris data**

| **GO terms enriched in the largest connected component in T cells** | | |
| --- | --- | --- |
| ID | Description | Adjusted *P* |
| GO:0006412 | translation | 7.22E-55 |
| GO:0043043 | peptide biosynthetic process | 7.22E-55 |
| GO:0043604 | amide biosynthetic process | 2.46E-54 |
| GO:0006518 | peptide metabolic process | 5.63E-50 |
| GO:0043603 | cellular amide metabolic process | 6.44E-49 |
| GO:1901566 | organonitrogen compound biosynthetic process | 6.54E-40 |
| GO:0034645 | cellular macromolecule biosynthetic process | 3.07E-23 |
| GO:0042254 | ribosome biogenesis | 8.85E-23 |
| GO:0009059 | macromolecule biosynthetic process | 4.46E-22 |
| GO:0044271 | cellular nitrogen compound biosynthetic process | 6.60E-21 |
| **GO terms enriched in the largest connected component in muscle cells** | | |
| ID | Description | Adjusted *P* |
| GO:0006412 | translation | 2.35E-41 |
| GO:0043043 | peptide biosynthetic process | 5.35E-41 |
| GO:0043604 | amide biosynthetic process | 1.56E-40 |
| GO:0006518 | peptide metabolic process | 1.76E-37 |
| GO:0043603 | cellular amide metabolic process | 4.88E-37 |
| GO:1901566 | organonitrogen compound biosynthetic process | 1.52E-30 |
| GO:0044267 | cellular protein metabolic process | 8.28E-18 |
| GO:0009059 | macromolecule biosynthetic process | 1.11E-17 |
| GO:0034645 | cellular macromolecule biosynthetic process | 1.32E-17 |
| GO:0019538 | protein metabolic process | 7.94E-17 |
| **GO terms enriched in the largest connected component in beta cells** | | |
| ID | Description | Adjusted *P* |
| GO:0034976 | response to endoplasmic reticulum stress | 4.25E-03 |

*Note*: A significance level of 0.01 was applied to the FDR-adjusted *P* values. Only the most significant 10 GO terms were shown if more than 10 were enriched.
