# Supplementary material for: scLink: Inferring Sparse Gene Co-expression Networks from Single-cell Expression Data
Source: Genomics Proteomics Bioinformatics. 2021 Jul 10;19(3):475–92. doi: 10.1016/j.gpb.2020.11.006 (PMC8896229; doi:10.1016/j.gpb.2020.11.006)
Supplement: Supplementary Table S6 — GO terms enriched in the largest connected components of glasso-f networks constructed from Tabula Muris data [file mmc23.docx]

**Table S6 GO terms enriched in the largest connected components of glasso-f networks constructed from Tabula Muris data**

| **GO terms enriched in the largest connected component in T cells** | | |
| --- | --- | --- |
| ID | Description | Adjusted *P* |
| GO:0006457 | protein folding | 6.230E-07 |
| GO:1901998 | toxin transport | 4.857E-03 |
| GO:0006413 | translational initiation | 6.302E-03 |
| GO:0019882 | antigen processing and presentation | 6.302E-03 |
| GO:0010499 | proteasomal ubiquitin-independent protein catabolic process | 6.302E-03 |
| GO:0006278 | RNA-dependent DNA biosynthetic process | 6.302E-03 |
| GO:0007004 | telomere maintenance via telomerase | 6.302E-03 |
| GO:2000573 | positive regulation of DNA biosynthetic process | 6.302E-03 |
| GO:0010498 | proteasomal protein catabolic process | 6.302E-03 |
| GO:0043161 | proteasome-mediated ubiquitin-dependent protein catabolic process | 8.453E-03 |
| **GO terms enriched in the largest connected component in muscle cells** | | |
| ID | Description | Adjusted *P* |
| GO:0051254 | positive regulation of RNA metabolic process | 3.82E-08 |
| GO:0045893 | positive regulation of transcription, DNA-templated | 7.16E-08 |
| GO:1902680 | positive regulation of RNA biosynthetic process | 7.16E-08 |
| GO:1903508 | positive regulation of nucleic acid-templated transcription | 7.16E-08 |
| GO:0045935 | positive regulation of nucleobase-containing compound process | 1.24E-07 |
| GO:0006357 | regulation of transcription by RNA polymerase II | 2.04E-07 |
| GO:0006366 | transcription by RNA polymerase II | 2.04E-07 |
| GO:0045944 | positive regulation of transcription by RNA polymerase II | 2.04E-07 |
| GO:0010557 | positive regulation of macromolecule biosynthetic process | 1.53E-06 |
| GO:0031328 | positive regulation of cellular biosynthetic process | 1.62E-06 |
| **GO terms enriched in the largest connected component in beta cells** | | |
| ID | Description | Adjusted *P* |
| GO:0006518 | peptide metabolic process | 9.648E-11 |
| GO:0006412 | translation | 2.542E-10 |
| GO:0043043 | peptide biosynthetic process | 3.214E-10 |
| GO:0043603 | cellular amide metabolic process | 3.515E-10 |
| GO:0043604 | amide biosynthetic process | 1.144E-09 |
| GO:1901566 | organonitrogen compound biosynthetic process | 5.779E-05 |
| GO:0022618 | ribonucleoprotein complex assembly | 5.779E-05 |
| GO:0042255 | ribosome assembly | 5.839E-05 |
| GO:0002181 | cytoplasmic translation | 1.321E-04 |
| GO:0022613 | ribonucleoprotein complex biogenesis | 2.485E-04 |

*Note*: A significance level of 0.01 was applied to the FDR-adjusted *P* values. Only the most significant 10 GO terms were shown if more than 10 were enriched.
