# Supplementary material for: scLink: Inferring Sparse Gene Co-expression Networks from Single-cell Expression Data
Source: Genomics Proteomics Bioinformatics. 2021 Jul 10;19(3):475–92. doi: 10.1016/j.gpb.2020.11.006 (PMC8896229; doi:10.1016/j.gpb.2020.11.006)
Supplement: Supplementary Table S7 — GO terms enriched in the largest connected components of glasso-r networks constructed from Tabula Muris data [file mmc24.docx]

**Table S7 GO terms enriched in the largest connected components of glasso-r networks constructed from Tabula Muris data**

| **GO terms enriched in the largest connected component in T cells** | | |
| --- | --- | --- |
| ID | Description | Adjusted *P* |
| GO:0010498 | proteasomal protein catabolic process | 1.95E-07 |
| GO:0010499 | proteasomal ubiquitin-independent protein catabolic process | 1.38E-06 |
| GO:0051603 | proteolysis involved in cellular protein catabolic process | 2.07E-06 |
| GO:0044257 | cellular protein catabolic process | 4.37E-06 |
| GO:0043161 | proteasome-mediated ubiquitin-dependent protein catabolic process | 4.69E-06 |
| GO:0044265 | cellular macromolecule catabolic process | 5.41E-06 |
| GO:0006508 | proteolysis | 2.19E-05 |
| GO:0030163 | protein catabolic process | 2.19E-05 |
| GO:0006511 | ubiquitin-dependent protein catabolic process | 2.19E-05 |
| GO:1901565 | organonitrogen compound catabolic process | 2.29E-05 |
| **GO terms enriched in the largest connected component in muscle cells** | | |
| ID | Description | Adjusted *P* |
| GO:0051254 | positive regulation of RNA metabolic process | 6.95E-08 |
| GO:0006357 | regulation of transcription by RNA polymerase II | 6.95E-08 |
| GO:0006366 | transcription by RNA polymerase II | 6.95E-08 |
| GO:0010628 | positive regulation of gene expression | 6.95E-08 |
| GO:2000112 | regulation of cellular macromolecule biosynthetic process | 1.60E-07 |
| GO:0010556 | regulation of macromolecule biosynthetic process | 2.14E-07 |
| GO:0031326 | regulation of cellular biosynthetic process | 2.14E-07 |
| GO:0009889 | regulation of biosynthetic process | 3.05E-07 |
| GO:0045935 | positive regulation of nucleobase-containing compound process | 3.39E-07 |
| GO:0018130 | heterocycle biosynthetic process | 7.05E-07 |
| **GO terms enriched in the largest connected component in beta cells** | | |
| ID | Description | Adjusted *P* |
| GO:0006412 | Translation | 6.24E-12 |
| GO:0043043 | peptide biosynthetic process | 6.24E-12 |
| GO:0006518 | peptide metabolic process | 6.24E-12 |
| GO:0043604 | amide biosynthetic process | 2.27E-11 |
| GO:0043603 | cellular amide metabolic process | 3.55E-11 |
| GO:1901566 | organonitrogen compound biosynthetic process | 1.17E-07 |
| GO:0034645 | cellular macromolecule biosynthetic process | 3.30E-06 |
| GO:0042255 | ribosome assembly | 3.43E-06 |
| GO:0009059 | macromolecule biosynthetic process | 4.24E-06 |
| GO:0044267 | cellular protein metabolic process | 1.83E-05 |

*Note*: A significance level of 0.01 was applied to the FDR-adjusted *P* values. Only the most significant 10 GO terms were shown if more than 10 were enriched.
