# Supplementary material for: scLink: Inferring Sparse Gene Co-expression Networks from Single-cell Expression Data
Source: Genomics Proteomics Bioinformatics. 2021 Jul 10;19(3):475–92. doi: 10.1016/j.gpb.2020.11.006 (PMC8896229; doi:10.1016/j.gpb.2020.11.006)
Supplement: Supplementary Table S8 — GO enrichment analysis on genes with large degree changes between normal and breast cancer conditions [file mmc25.docx]

**Table S8 GO enrichment analysis on genes with large degree changes between normal and breast cancer conditions**

| **scLink networks** | | |
| --- | --- | --- |
| ID | Description | Adjusted *P* |
| GO:0002444 | myeloid leukocyte mediated immunity | 7.39E-05 |
| GO:0002446 | neutrophil mediated immunity | 0.0001 |
| GO:0043299 | leukocyte degranulation | 0.0001 |
| GO:0002275 | myeloid cell activation involved in immune response | 0.0001 |
| GO:0043312 | neutrophil degranulation | 0.0003 |
| GO:0002283 | neutrophil activation involved in immune response | 0.0003 |
| GO:0042119 | neutrophil activation | 0.0003 |
| GO:0036230 | granulocyte activation | 0.0003 |
| GO:0042060 | wound healing | 0.0054 |
| GO:0007596 | blood coagulation | 0.0088 |
| **Pearson correlation networks** | | |
| ID | Description | Adjusted *P* |
| GO:0030449 | regulation of complement activation | 3.80E-06 |
| GO:2000257 | regulation of protein activation cascade | 3.80E-06 |
| GO:0002920 | regulation of humoral immune response | 1.34E-05 |
| GO:0006956 | complement activation | 1.48E-05 |
| GO:0072376 | protein activation cascade | 2.57E-05 |
| GO:0002673 | regulation of acute inflammatory response | 3.14E-05 |
| GO:0070613 | regulation of protein processing | 6.72E-05 |
| GO:1903317 | regulation of protein maturation | 6.72E-05 |
| GO:0006958 | complement activation, classical pathway | 7.06E-05 |
| GO:0002455 | humoral immune response mediated by circulating immunoglobulin | 0.0002 |

*Note*: A significance level of 0.01 was applied to the FDR-adjusted *P* values. Only the most significant 10 GO terms were shown if more than 10 were enriched.
